# Lactylation-driven transcriptional activation of FBXO33 promotes gallbladder cancer metastasis by regulating p53 polyubiquitination

**DOI:** 10.1038/s41419-025-07372-y

**Published:** 2025-02-28

**Authors:** Zhenheng Wu, You Peng, Wen Chen, Feng Xia, Tieshan Song, Qiming Ke

**Affiliations:** 1 Department of Hepatobiliary Surgery and Fujian Institute of Hepatobiliary Surgery, Fujian Medical University Union Hospital, Fuzhou, 350001 Fujian China; 2 The Third Affiliated Hospital of Sun Yat-sen University, Zhaoqing Hospital, Health Management Center, Zhaoqing, 526070 Guangdong China; 3 Department of Hepatobiliary Surgery, Fuzhou First Hospital Affiliated with Fujian Medical University, Fuzhou, 350009 Fujian China; 4 Department of Hepatic Surgery Center, Tongji Hospital of Tongji Medical College of Huazhong University of Science and Technology, Wuhan, China; 5 The Basic Medical School, Hubei University of Science and Technology, Xianning, 437100 Hubei China; 6 Department of General Surgery, Tianjin Medical University General Hospital, Tianjin, 300052 China

**Keywords:** Gall bladder cancer, Ubiquitylation

## Abstract

Gallbladder cancer (GBC) is the most common malignant tumor of the biliary tract and is often prone to early distant metastasis. However, the mechanisms underlying GBC’s invasive metastasis remain unclear. This study identified that F-box only protein 33 (FBXO33) expression is significantly elevated in GBC and is negatively associated with patient prognosis. In vivo and in vitro experiments demonstrated that knockdown of FBXO33 inhibits epithelial-mesenchymal transition (EMT) progression in GBC, while overexpression of FBXO33 promotes EMT progression. Mechanistically, FBXO33 regulates EMT progression by modulating the polyubiquitination of p53 at K291 and K292. Moreover, the upregulation of FBXO33 in GBC is driven by transcriptional regulation mediated by Yin Yang-1 (YY1). The lactylation modification of YY1 at K183 was found to be essential for the transcriptional activation of FBXO33. These findings underscore the role of the lactylation-driven FBXO33-p53 axis in promoting the invasive metastasis of GBC.

## Introduction

GBC is the most common malignant tumor of the biliary tract, characterized by a high mortality rate and poor prognosis [1, 2]. Despite extensive research, no significant improvement in GBC prognosis has been achieved [3]. Early metastasis is a hallmark of GBC, often making surgical treatment unfeasible at diagnosis [4]. Thus, identifying effective early biomarkers and therapeutic targets for GBC is essential. Studies suggest that EMT plays a critical role in initiating distant metastasis in malignant tumors [5]; however, the molecular mechanisms driving EMT in GBC remain unclear. This highlights an urgent need to identify novel EMT-related biomarkers to improve patient outcomes.

Protein ubiquitination involves the covalent attachment of a 76-amino-acid ubiquitin protein to target proteins, mediated by ubiquitin-activating enzymes (E1), ubiquitin-conjugating enzymes (E2), and ubiquitin ligases (E3) [6, 7]. This process regulates the biological functions of target proteins and is integral to key signaling pathways, influencing metastasis and treatment responses in malignant tumors [8, 9]. The F-box family of E3 ubiquitin ligases has been shown to play a pivotal role in EMT progression [10]. For example, FBXO11 targets Snail for ubiquitination and degradation, thereby inhibiting EMT and metastasis in breast cancer models [11], and also regulates EMT via ubiquitination of SAMD1 [12]. Conversely, FBXO32 facilitates EMT by ubiquitinating and degrading substrates such as CTBP1 and PTEN [13, 14]. Recent studies have identified that FBXO33, a member of the F-box family, acts as a tumor suppressor by promoting ubiquitination and degradation of MYC, thereby inhibiting metastasis in non-small cell lung cancer [15]. However, the role of FBXO33 in other tumor types, including GBC, remains unclear.

This study demonstrates that FBXO33, a novel E3 ubiquitin ligase, is significantly upregulated in GBC. The effects of FBXO33 on EMT in GBC and its underlying mechanisms were evaluated. The findings indicate that FBXO33 functions as an oncogene in GBC. Overexpression of FBXO33 promotes EMT progression, while its downregulation inhibits EMT progression. Mechanistically, ubiquitination at K291 and K292 of p53 is critical for FBXO33-mediated regulation of EMT in GBC. Furthermore, transcriptional activation of FBXO33 in GBC depends on regulation by the transcription factor YY1. This activation is further reliant on the lactylation modification at K183 of the YY1 protein.

## Materials and methods

### Patient data collection

Clinical samples were collected in strict accordance with the approved protocol by the Ethics Committee of Fujian Medical University’s Medical College, China. Eighty-two paraffin-embedded tumor specimens diagnosed as GBC by the Clinical Pathology Department of Fujian Medical University Union Hospital from January 2013 to January 2024, along with paired benign tissues, were collected. Informed consent was obtained from all patients. None of the patients received any radiotherapy or chemotherapy prior to tumor resection.

### Cell lines and cell culture

Human GBC cell lines GBC-SD and SGC-996 were purchased from the Shanghai Institute of Life Sciences, China. The 293T cell line was provided by the Department of Oncology, Tongji University School of Medicine, China. All cell lines underwent mycoplasma detection and STR cell identification to ensure their authenticity and purity. All cells were maintained in high-glucose Dulbecco’s Modified Eagle Medium (DMEM, Hyclone, USA) supplemented with 10% fetal bovine serum (FBS, Hyclone, USA) and cultured at 37 °C in a humidified atmosphere containing 5% CO2. All cells were tested negative for mycoplasma contamination.

### Next-generation sequencing

Three random 0.8 mm tumor tissue cores were selected to collect tumor DNA. For samples with low tumor volume, 5-10 tissue slides were used to microdissect the selected tumor regions for DNA enrichment. Although DNA was isolated from the same tumor block, the p53 IHC slides were not used to guide the microdissection areas. The samples were sequenced using the AmpliSeq Cancer Hotspot Panel version 5. All p53 mutation calls were cross-referenced against publicly available databases (ClinVar, available at https://www.ncbi.nlm.nih.gov/clinvar/ and COSMIC, available at https://cancer.sanger.ac.uk/cosmic) [16, 17]. The criteria for mutation reporting were a minimum read depth of 500, an allele ratio ≥5%, a base quality score ≥30, a probability score for single nucleotide changes ≥0.90, and a quality score for insertions/deletions ≥1000.

### Real-time quantitative polymerase chain reaction (RT-qPCR)

Total RNA was extracted from GBC tissues or cells using TRIzol (15596-026, Invitrogen, USA). mRNA was reverse transcribed using the All-In-One 5×RT MasterMix (Abm, Canada). Subsequently, PCR was performed using Fast Start Universal SYBR Green Master Mix (Roche, Basel, Switzerland) and fluorescence quantification was conducted according to the operation manual of the ABI 7500 real-time system. GAPDH was used as an internal control for normalization. The relative expression levels of mRNA were evaluated using the 2 − ΔΔCt method. Detailed information on primer sequences is provided in Table 1.

**Table 1:** Detailed information of the primer sequences in this study.

Gene	Primer sequence
FBXO33	F 5’-ATGTGCCTTTGCAACGACTG-3’
	R 5’-TGACAGGGCTTTCCAATGCT-3’
P53	F 5’-CAGCACATGACGGAGGTTGT-3’
	R 5’-TCATCCAAATACTCCACACGC-3’
GAPDH	F 5’-GGTGTGAACCATGAGAAGTATGA-3’
	R 5’-GAGTCCTTCCACGATACCAAAG-3’

### Western blot analysis (WB) and antibodies

Total protein was extracted from GBC tissues or cells using pre-cooled RIPA (Radioimmunoprecipitation assay) buffer (Beyotime, Shanghai, China). Subsequently, the protein lysates were quantified using the BCA (Bicinchoninic acid) protein assay kit (Thermo Fisher Scientific). After separating the proteins by SDS-PAGE electrophoresis, they were transferred onto PVDF membranes. The membranes were then blocked with 5% non-fat milk for 2 h at room temperature and incubated with primary antibodies overnight at 4 °C. After incubation with secondary antibodies at room temperature for 1 h, the antibody-antigen complexes were detected using the ECL assay kit (Advansta, USA). The following primary antibodies were purchased: rabbit anti-YY1 (ab227269), rabbit anti-YY1 (ab109237), rabbit anti-p53 (ab32049), mouse anti-p53 (ab308609), rabbit anti-Flag (ab205606), mouse anti-Flag (ab125243), rabbit anti-His (ab213204), mouse anti-His (ab18184), rabbit anti-HA (ab9110), rabbit anti p65(ab32536), rabbit anti Myc-tag(ab9106), mouse anti Myc-tag(ab32), rabbit anti-Ubiquitin (ab134953) from Abcam; lactylation pan-antibody (PTM-1401RM) from PTM BIO (Hangzhou, China); rabbit anti-FBXO33 (PA5-61275) from Thermo Fisher Scientific; rabbit anti-E-Cadherin (#3195), rabbit anti-Vimentin (#5741), rabbit secondary antibody (#7074), and mouse secondary antibody (#7076) from Cell Signaling Technology. Rabbit anti-GAPDH (#5174) was used as an internal control for normalization.

### Chemical inhibitors

The following exogenous drugs used in this study were purchased from MCE (MedChemExpress, USA): MG132 (HY-13259), Cycloheximide (CHX, HY-12320), and Lactate (HY-B2227).

### Immunohistochemical analysis (IHC)

Paraffin-embedded sections were deparaffinized in xylene, dehydrated in graded ethanol, and subjected to antigen retrieval. The sections were washed with TBS-T and quenched in a 3% hydrogen peroxide TBS solution for 10 minutes to block endogenous peroxidase/biotin activity. Non-specific binding was blocked with a serum-free blocking reagent, followed by overnight incubation with primary antibodies. Primary antibodies specific for FBXO33 (1:500, Thermo Fisher Scientific), p53 (1:400, Abcam), E-Cadherin (1:200, #3195), and Vimentin (1:300, #5741) were incubated overnight at 4 °C. The sections were then incubated with secondary antibodies for 20 min at room temperature in a humid chamber. Visualization was achieved using DAB (3,3’-Diaminobenzidine) solution, and the sections were counterstained with hematoxylin. Digital pathology slide scanner (Olympus VS200, Japan) was used to scan the images, and Image-Pro Plus 6.0 (Media Cybernetics, USA) was employed for analysis. The quantification of immunohistochemical evaluation was based on the product of staining intensity and the percentage of positively stained cells. The percentage of positively stained cells was scored on a scale from 0 to 4 (0 = 0–10%; 1 = 11–25%; 2 = 26–50%; 3 = 51–75%; 4 = 76–100%), while staining intensity was rated on a scale from 0 to 3 (0 = no staining; 1 = weak; 2 = moderate; 3 = strong). The overall protein expression in each sample was expressed as histoscore, which was multiplication product of the staining intensity score(0–3) and percentage of positively stained cells score(0–4) and is between 0 and 12. Low expression is scored 0–4. High expression is scored 6–12. The staining score was evaluated by two independent pathologists.

### Lentivirus construction and transfection

Lentiviral particles carrying Flag-tagged FBXO33-GFP (Flag-FBXO33) and its control vector lentivirus (Vector 1), as well as lentiviral particles containing the YY1 gene coding region for overexpression (OE-YY1) and its control vector lentivirus (Vector 2), were obtained from GENE (Shanghai, China). GBC cells were infected with lentivirus for 24 hours. Stable clones were selected with puromycin (5 μg/ml; Sigma). Lentivector-mediated short-hairpin FBXO33 (sh-FBXO33) and non-targeting plasmids (sh-Scr 1) and lentivector-mediated short-hairpin YY1 (sh-YY1) and non-targeting plasmids (sh-Scr 2)were designed and synthesized by GENE (Shanghai, China). Stable transfer GBC cells with FBXO33 and YY1 knockdown were generated in the same way.

### Plasmid transfection

All plasmids used for cell transfection were obtained from GENE (Shanghai, China). Cells were seeded in 6-well plates or 10 cm^2^ culture dishes. When cells reached 70–80% confluence, transfection was performed using Lipofectamine 3000 reagent (Invitrogen Thermo Fisher Scientific, USA) according to the manufacturer’s instructions. Cells were collected 48 h post-transfection for subsequent experiments.

### Generation of p53-knockout (p53-KO) GBC cell lines

p53-knockout GBC cell lines were constructed using the CRISPR–Cas9 gene-editing system. Lentiviruses containing Cas9-guide RNA targeting sequences were designed and synthesized by GENE (Shanghai, China). Lentivirus infection was performed on GBC cells at 80% confluency. The cells were selected after culture for one week in a medium containing 5 μg/ml puromycin. Monocolonies were picked, and the knockout efficiency was determined by western blotting. The sequence of single guide RNAs (sgRNAs) targeting human p53: 5ʹ-CCATTGTTCAATATCGTCCG-3ʹ and 5ʹ-CGGACGATATTGAACAATGG-3ʹ. The non-specific control sgRNA in the same vector were defined as Vector-KO cells.

### Dual-luciferase reporter assay

Bioinformatics analysis based on the JASPAR database indicated binding sites for YY1 in the FBXO33 promoter region. Plasmids containing the wild-type (WT) and mutant (MUT) FBXO33 promoter regions were obtained from GENE (Shanghai, China) based on the sequences of these binding sites. The WT and MUT FBXO33 promoter region plasmids were separately transfected into different experimental and corresponding control group cells. After 48 h, firefly luciferase (F-luc) activity was measured using the Dual-Luciferase Reporter Assay System (Promega E2920, USA) according to the manufacturer’s instructions, with Renilla luciferase (R-luc) used as an internal control for normalization.

### Xenograft tumor model

Female athymic BALB/c nude mice (4–6 weeks old) were purchased from SIPEIFU (Beijing, China). All mice were housed under specific pathogen-free conditions, strictly in accordance with the approved protocol of the Ethical Committee of Fujian Medical University’s Medical College (Approval No: IACUC FJMU 2023-0197).

Mice were randomly divided into experimental groups. GBC SGC-996 cells (sh-FBXO33 group and corresponding sh-Scr group (4–6 weeks old, *n* = 6) or Flag-FBXO33 group and corresponding Vector group (4–6 weeks old, *n* = 5), 1 × 10^7^ cells per group) were suspended in 100 μl of sterile PBS. The cells were subcutaneously implanted into the right hind limb of the mice using a syringe to establish the xenograft tumor model. Tumor growth was monitored weekly, and tumor volume was calculated using the formula: (length × width^2^)/2. After 4 weeks of tumor formation, the mice were euthanized, and xenograft specimens were collected by surgical excision and weighed.

### Protein stability

CHX (final concentration 50 μg/ml) was added to the cells. After specified incubation times, protein levels of p53 were assessed using western blotting.

### Chromatin immunoprecipitation (ChIP-qPCR) analysis

The ChIP assay was conducted using a SimpleChIP Plus Enzymatic Chromatin IP Kit (CST, 9004S). Briefly, GBC cells (1 × 10^7^ cells per group) were prepared according to the kit instructions and resuspended in ChIP buffer. Chromatin DNA fragments were enzymatically sheared, and antibody-magnetic bead complexes were used to pull down DNA fragments at 4 °C. Target DNA fragments were then detected using RT-qPCR.

### Co-Immunoprecipitation (Co-IP)

When cell confluency reached 80–90% in 10 cm^2^ dishes, Co-IP was performed according to the instructions of the protein immunoprecipitation kit (Geneseed, Guangzhou, China). In brief, cell lysates were prepared and protein concentrations were quantified using BCA protein assay. Subsequently, 1 mg of total protein was incubated overnight at 4 °C with antibody-magnetic bead complexes. The following day, antibody-antigen complexes were eluted, and subsequent protein blotting was performed for relevant detection. Anti-rabbit IgG for IP (RA1009-01) was purchased from Vazyme (Nanjing, China) to prevent detection of IP antibody heavy chains (55 kDa).

### Statistical analysis

In this study, all statistical analyses were performed using SPSS 19.0 software package (SPSS Inc., Chicago, USA) and GraphPad Prism 8 software (GraphPad, USA). Clinical pathological data analysis employed chi-square analysis and Pearson correlation analysis. For two-group comparisons, we employed the t-test to evaluate the significance of differences. When analyzing differences among multiple groups, we utilized analysis of variance (One-way ANOVA). All data are presented as mean ± standard error of the mean (SEM) of independent replicates (*n* ≥ 3). Differences with *p* < 0.05 were considered statistically significant.

## Results

### High expression of FBXO33 in GBC is negatively correlated with overall survival in GBC

**Fig. 1:**
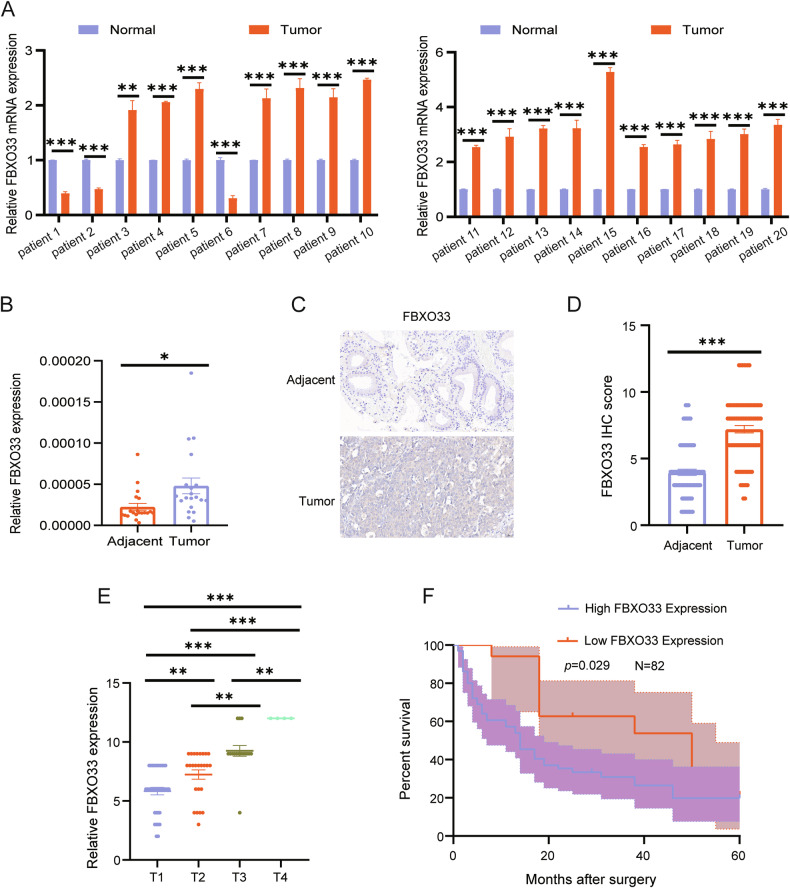
High expression of FBXO33 in GBC is negatively correlated with overall survival in GBC. **A**, **B** RT-qPCR was conducted to examine the expression of FBXO33 mRNA in 20 cases of GBC tissues and corresponding adjacent noncancerous tissues. **C**, **D** IHC was performed to assess the expression of FBXO33 in 82 cases of GBC tissues and corresponding adjacent noncancerous tissues. **E** Expression of FBXO33 in GBC patients at different stages. **F** Relationship between FBXO33 expression and overall survival rate of GBC patients. Error bars represent the mean (*n* = 3) ±SEM. *P < 0.05, **P < 0.01, ***P < 0.001.

To investigate the role of FBXO33 in GBC, mRNA expression levels of FBXO33 were measured in 20 paired GBC tumor and adjacent gallbladder tissues using RT-qPCR. The results revealed that FBXO33 expression was significantly higher in GBC tissues compared to adjacent tissues (Fig. 1A, B). Additionally, IHC staining was performed to assess protein expression levels of FBXO33 in 82 paired GBC tumor and adjacent tissues. The findings confirmed that FBXO33 expression was elevated in cancer tissues compared to adjacent tissues (Fig. 1C, D). FBXO33 expression was positively associated with distant metastasis and TNM staging but showed no significant correlation with gender, age, or the presence of gallstones (Table 2). Moreover, FBXO33 expression progressively increased from T1 to T4 stages of GBC (Fig. 1E). Kaplan-Meier survival analysis demonstrated that GBC patients with low FBXO33 expression had higher overall survival rates compared to those with high FBXO33 expression (Fig. 1F). These results indicate that FBXO33 acts as an oncogene and is highly expressed in GBC.

**Table 2:** Correlation analysis of FBXO33 expression and clinical pathological features in GBC.

Clinicopathological features		Protein expression of FBXO33		
	Case	High (*n* = 65)	Low (*n* = 17)	*P*
Age (years)				0.799
<60 years old	46	36	10	
≥60 years old	36	29	7	
Gender				0.088
Male	38	27	11	
Female	44	38	6	
TNM stage				0.046*
I–II	62	46	16	
III–IV	20	19	1	
Distant metastasis				0.024*
(+)	44	39	5	
(−)	38	26	12	
Gallstone				0.768
(+)	46	37	9	
(−)	36	28	8	

Low FBXO33 expression is scored 0–4.

High FBXO33 expression is scored 6–12.

Bold value indicates significant differences between groups.

*P < 0.05.

### FBXO33 regulates the progression of EMT in GBC in vivo and in vitro

**Fig. 2:**
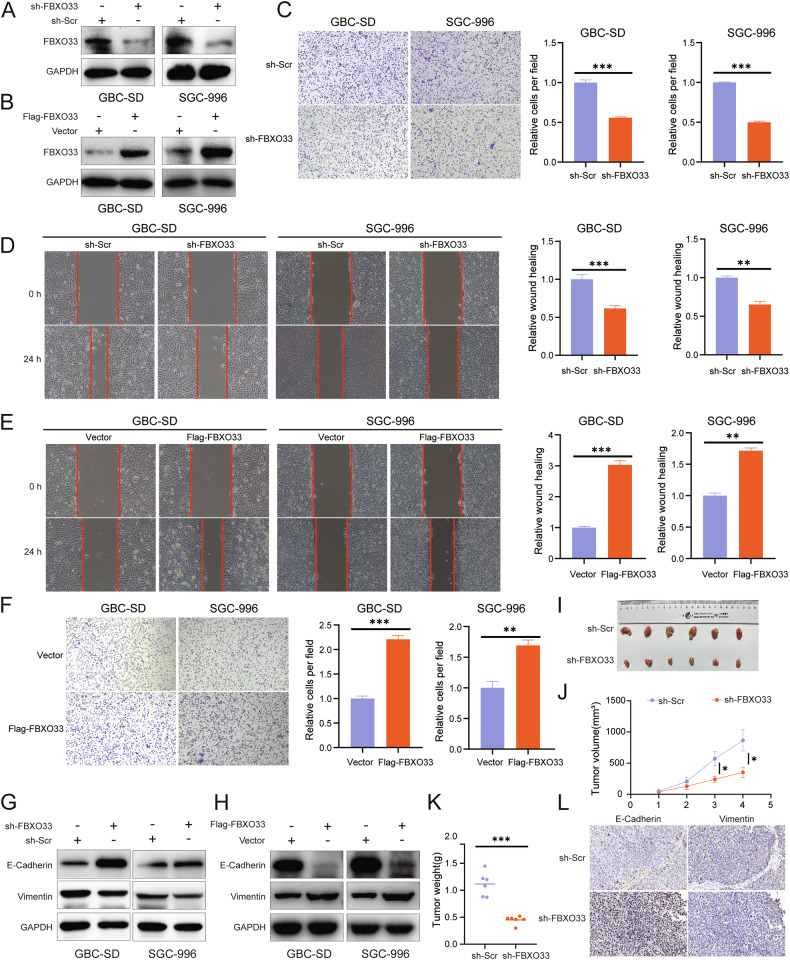
FBXO33 regulates EMT progression of GBC in vitro and in vivo. **A** Western blot was used to detect the knockdown effect of sh-FBXO33. **B** Western blot was utilized to examine the overexpression effect of Flag-FBXO33. **C**, **D** Transwell chamber assay and scratch assay were performed to evaluate the impact of sh-FBXO33 on the migration ability of GBC cells. **E**, **F** Scratch assay and Transwell chamber assay were conducted to assess the effect of Flag-FBXO33 on the migration ability of GBC cells. **G** Western blot detected changes in E-Cadherin and Vimentin protein levels in GBC cells after FBXO33 knockdown. **H** Western blot detected changes in E-Cadherin and Vimentin protein levels in GBC cells after FBXO33 overexpression. **I** Impact of sh-FBXO33 on subcutaneous tumor size in GBC. Impact of sh-FBXO33 on subcutaneous tumor (**J**) weight and (**K**) volume in GBC. **L** IHC analysis of the effect of sh-FBXO33 on the protein expression of E-Cadherin and Vimentin in GBC subcutaneous tumors. Error bars represent the mean (*n* = 3) ±SEM. *P < 0.05, **P < 0.01, ***P < 0.001.

To clarify the role of FBXO33 in GBC invasion and metastasis, GBC cells with FBXO33 knockdown (sh-FBXO33) or overexpression (Flag-FBXO33) were constructed using lentiviral vectors (Fig. 2A, B). Scratch and Transwell assays revealed that cell migration was significantly reduced in the sh-FBXO33 group compared to the sh-Scr control group (Fig. 2C, D), while migration was significantly enhanced in the Flag-FBXO33 group compared to the Vector control group (Fig. 2E, F). Western blot analysis showed that in the sh-FBXO33 group, E-Cadherin expression was significantly upregulated, and Vimentin expression was significantly downregulated compared to the sh-Scr group (Fig. 2G and Supplementary Fig. S1A). Conversely, in the Flag-FBXO33 group, E-Cadherin expression was significantly downregulated, and Vimentin expression was significantly upregulated compared to the Vector group (Fig. 2H and Supplementary Fig. S1B).

To further validate these findings, a subcutaneous xenograft model of GBC was established using SGC-996 cells with either FBXO33 knockdown or overexpression and their respective control cells. Tumors in the sh-FBXO33 group were smaller and lighter compared to those in the sh-Scr group, indicating that FBXO33 knockdown inhibited GBC tumor growth (Fig. 2I–K). In contrast, tumors in the Flag-FBXO33 group were larger and heavier, suggesting that FBXO33 overexpression promoted tumor growth (Supplementary Fig. S1C–E). IHC analysis of subcutaneous xenografts confirmed that E-Cadherin expression was significantly increased and Vimentin expression significantly decreased in the sh-FBXO33 group compared to the sh-Scr group (Fig. 2L). Conversely, E-Cadherin expression was significantly reduced and Vimentin expression significantly elevated in the Flag-FBXO33 group compared to the Vector group (Supplementary Fig. S1F). In summary, these findings demonstrate that FBXO33 regulates the progression of EMT in GBC both in vivo and in vitro.

### FBXO33 interacts with p53

**Fig. 3:**
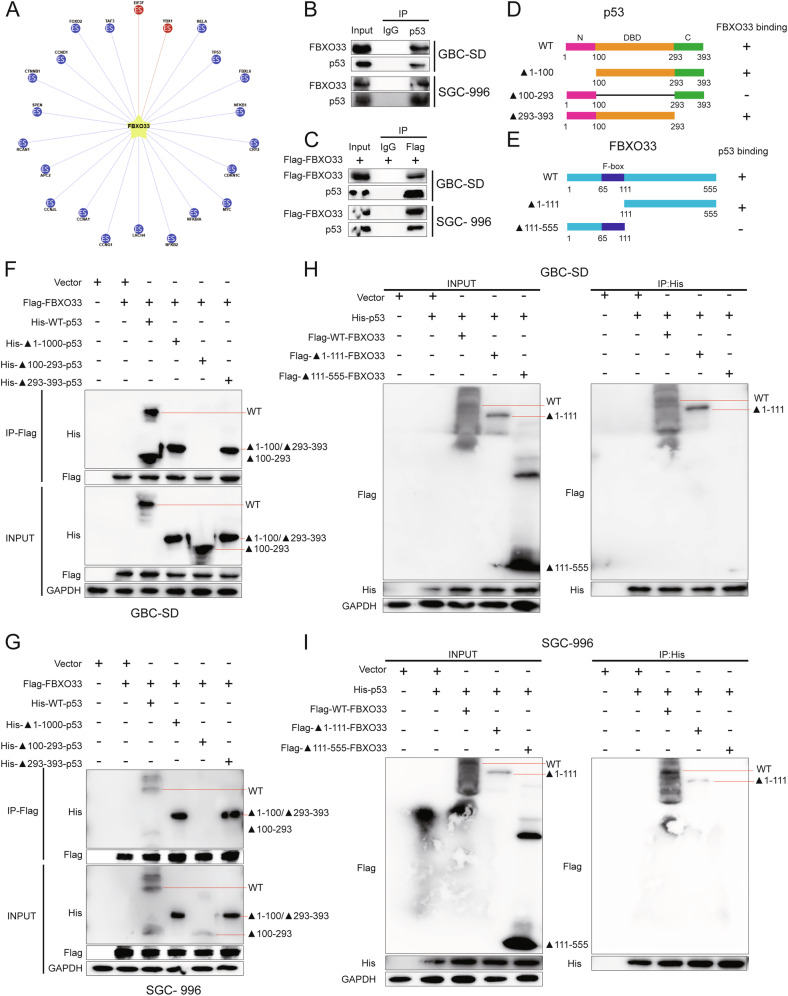
Interaction between FBXO33 and p53. **A** Bioinformatics databases predicted that p53 is a ubiquitination substrate of FBXO33 (The proteins marked in red are known substrates of FBXO33, while the proteins marked in blue are predicted substrates of FBXO33.). **B**, **C** Co-IP confirmed the binding of FBXO33 and p53. **D** Schematic diagram of p53 truncation construct. **E** Schematic diagram of FBXO33 truncation construct. **F**, **G** Co-IP confirmed the importance of the 100–293 domain of p53 protein for binding to p53 and FBXO33. **H**, **I** Co-IP confirmed the importance of the 111-555 domain of FBXO33 protein for binding to p53.

E3 ubiquitin ligases perform their biological functions by recognizing and regulating substrates [18], and FBXO33 is a member of this family. To understand the mechanism by which FBXO33 functions in GBC, the online tool Ubibrowser was used to predict potential substrates of FBXO33 [19]. Among the predicted substrates, RELA (p65) had the highest confidence score, followed by p53 (Fig. 3A and Supplementary Fig. S2A). Substrate ubiquitination is essential for understanding the function of E3 ubiquitin ligases, as well as their substrate recognition and regulatory roles [20]. To investigate the mechanism of FBXO33, Co-IP experiments were performed to assess the effect of Flag-FBXO33 on the ubiquitination of p65 and p53. The results showed that Flag-FBXO33 significantly increased the ubiquitination level of p53 compared to the Vector group, while it had minimal effect on p65 (Supplementary Fig. S2B, C). Thus, p53 was selected as the substrate of FBXO33 for further investigation. Co-IP experiments confirmed that FBXO33 binds to p53 (Fig. 3B, C). Given that many E3 ubiquitin ligases can target both wild-type p53 (wtp53) and mutant p53 (mutp53) [21], we investigated whether FBXO33 exhibits the same behavior. The p53 gene in GBC cell lines remained unmutated [22], indicating that the p53 interacting with FBXO33 in GBC-SD and SGC-996 cells is wtp53. Using CRISPR/Cas9, the endogenous p53 gene was knocked out in GBC-SD and SGC-996 cells (Supplementary Fig. S2D, E), followed by transfection with plasmids encoding Myc-tagged wtp53 and mutp53 variants. Co-IP experiments showed that Flag-FBXO33 significantly increased the ubiquitination level of wtp53 but had minimal effect on common mutp53 variants (p53R175H, p53R248W, and p53R273H) [23] (Supplementary Fig. S2F, G). Therefore, wtp53 was selected as the substrate of FBXO33 for further analysis.

To identify the interaction domains of FBXO33 and p53, truncated forms of p53 (WT-p53, ▲1-100-p53, ▲100–293-p53, ▲293-393-p53) (Fig. 3D) and FBXO33 (WT-FBXO33, ▲1-111-FBXO33, ▲111-555-FBXO33) were constructed (Fig. 3E). Co-IP results demonstrated that His-WT-p53, His-▲1-100-p53, and His-▲293-393-p53 could bind to Flag-FBXO33, while His-▲100–293-p53 lost this ability, indicating that the 100–293 domain of p53 is crucial for binding to FBXO33 (Fig. 3F, G). Similarly, Flag-WT-FBXO33 and Flag-▲1-111-FBXO33 could bind to His-p53, while Flag-▲111-555-FBXO33 lost this ability, showing that the 111-555 domain of FBXO33 is essential for binding to p53 (Fig. 3H, I). These findings confirm that FBXO33 interacts with p53.

### FBXO33 regulates the stability of the p53 protein

**Fig. 4:**
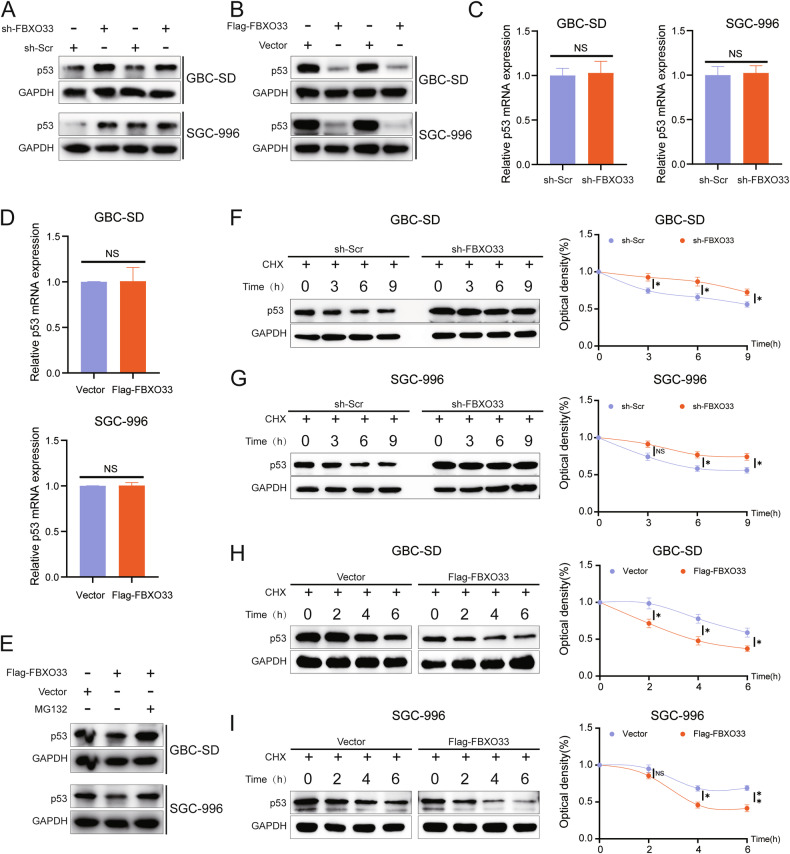
FBXO33 regulates the stability of p53 protein. **A** Western blot detected the effect of sh-FBXO33 on p53 protein levels in GBC cells. **B** Western blot detected the effect of Flag-FBXO33 on p53 protein levels in GBC cells. **C** RT-qPCR detected the effect of sh-FBXO33 on p53 mRNA levels in GBC cells. **D** RT-qPCR detected the effect of Flag-FBXO33 on p53 mRNA levels in GBC cells. **E** MG132 (20 uM) validated the degradation pathway of FBXO33 on p53 protein. **F**, **G** CHX (50 ug/ml) experiment examined the effect of sh-FBXO33 on the degradation rate of p53 protein. **H**, **I** CHX experiment assessed the effect of Flag-FBXO33 on the degradation rate of p53 protein. Error bars represent the mean (*n* = 3) ±SEM.^NS^*P* > 0.05, *P < 0.05, **P < 0.01.

Western blot analysis revealed that sh-FBXO33 significantly increased p53 protein expression (Fig. 4A and Supplementary Fig. S3A), while Flag-FBXO33 significantly decreased it (Fig. 4B and Supplementary Fig. S3B). RT-qPCR results indicated that neither sh-FBXO33 nor Flag-FBXO33 altered p53 mRNA expression (Fig. 4C, D), suggesting that FBXO33 regulates p53 protein expression at the post-translational level. Proteasome inhibitor MG132 alleviated the inhibitory effect of Flag-FBXO33 on p53, indicating that FBXO33 affects p53 protein expression through the ubiquitin-proteasome pathway (Fig. 4E and Supplementary Fig. S3C). To further evaluate the impact of FBXO33 on p53 protein stability, cycloheximide (CHX) was used to block protein synthesis. The CHX assay showed that p53 protein had a longer half-life in sh-FBXO33 GBC cells (Fig. 4F, G) and a shorter half-life in Flag-FBXO33 GBC cells (Fig. 4H, I). These results demonstrate that FBXO33 regulates p53 protein stability via the ubiquitin-proteasome pathway.

### Regulation of p53 ubiquitination by FBXO33

**Fig. 5:**
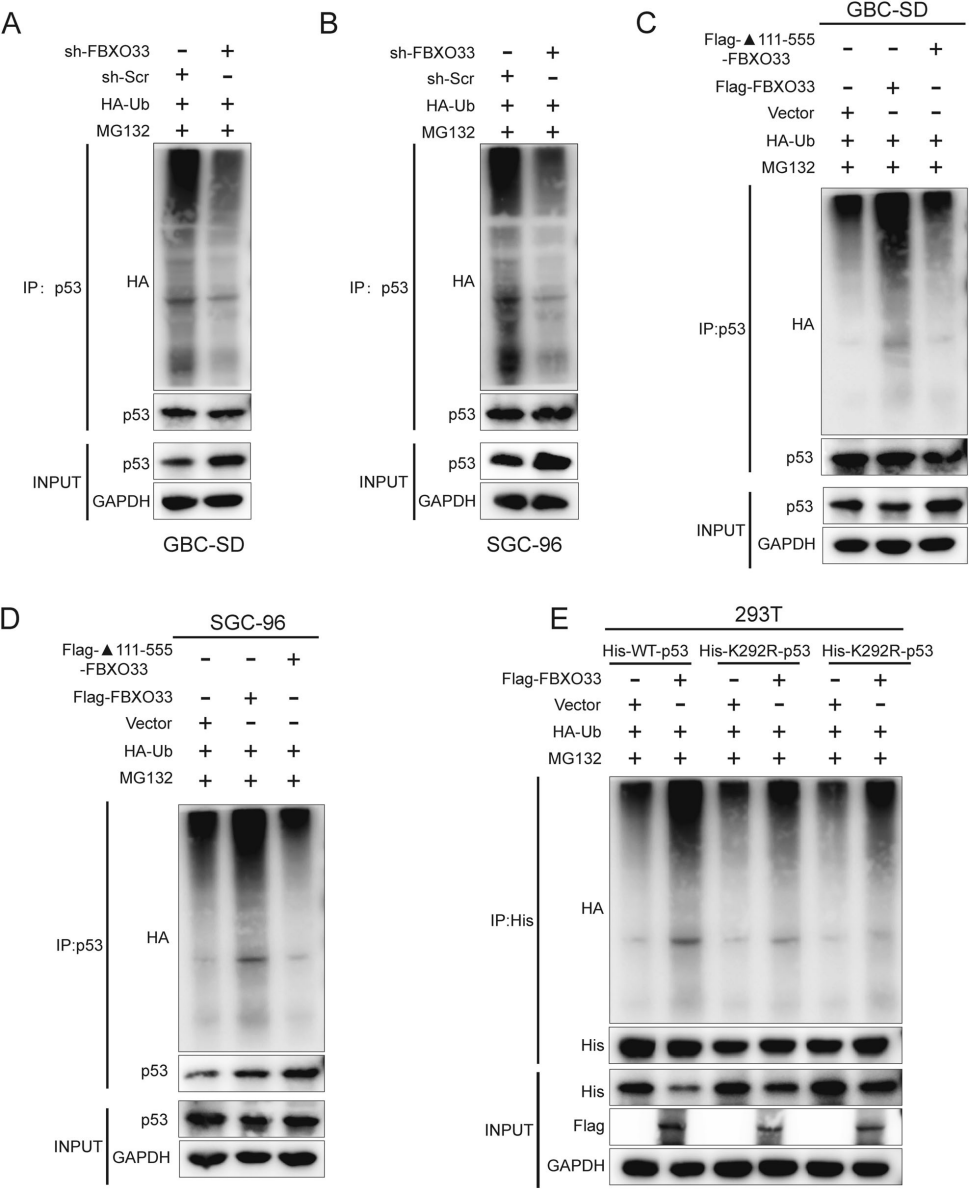
FBXO33 regulates ubiquitination modification of p53 at K291 and K292. **A**, **B** Co-IP detected the effect of sh-FBXO33 on the ubiquitin binding of p53. **C**, **D** Co-IP detected the ubiquitin binding of p53 after transfection of WT-FBXO33 and ▲111-555-FBXO33 into GBC cells. **E** His-WT-p53 or His-291R/292R-p53 was transfected into Flag-FBXO33 and corresponding vector group 293T cells. After 48 h, Co-IP was conducted to detect the ubiquitin binding of p53.

This study examined how FBXO33 regulates the stability of p53 protein. Co-IP assays were performed to assess endogenous p53 polyubiquitination levels. The results showed that sh-FBXO33 significantly reduced p53 polyubiquitination after MG132 treatment (Fig. 5A, B), whereas Flag-FBXO33 markedly increased p53 polyubiquitination (Supplementary Fig. S2B, C). Furthermore, deletion of the 111-555 domain of FBXO33 abolished its ability to inhibit p53 (Fig. 5C, D), indicating that this domain is essential for promoting p53 polyubiquitination.

Key ubiquitination sites within the 100–293 domain of p53 protein, lysine-291 (K291) and lysine-292 (K292), were identified [24]. To evaluate the roles of these sites in FBXO33-mediated p53 polyubiquitination, lysine-to-arginine mutations were introduced, resulting in His-K291R-p53 and His-K292R-p53 mutant plasmids. His-WT-p53, His-K291R-p53, and His-K292R-p53 were transfected into Flag-FBXO33 and Vector control cells. Western blot analysis revealed that K291R and K292R mutations reduced the inhibitory effect of Flag-FBXO33 on p53 protein (Fig. 5E). Co-IP experiments confirmed that these mutations weakened the promotion of p53 polyubiquitination by Flag-FBXO33 (Fig. 5E). These findings demonstrate that K291 and K292 are critical for FBXO33-mediated regulation of p53 protein.

### p53 regulates the function of FBXO33 in GBC and is negatively correlated with FBXO33 expression

**Fig. 6:**
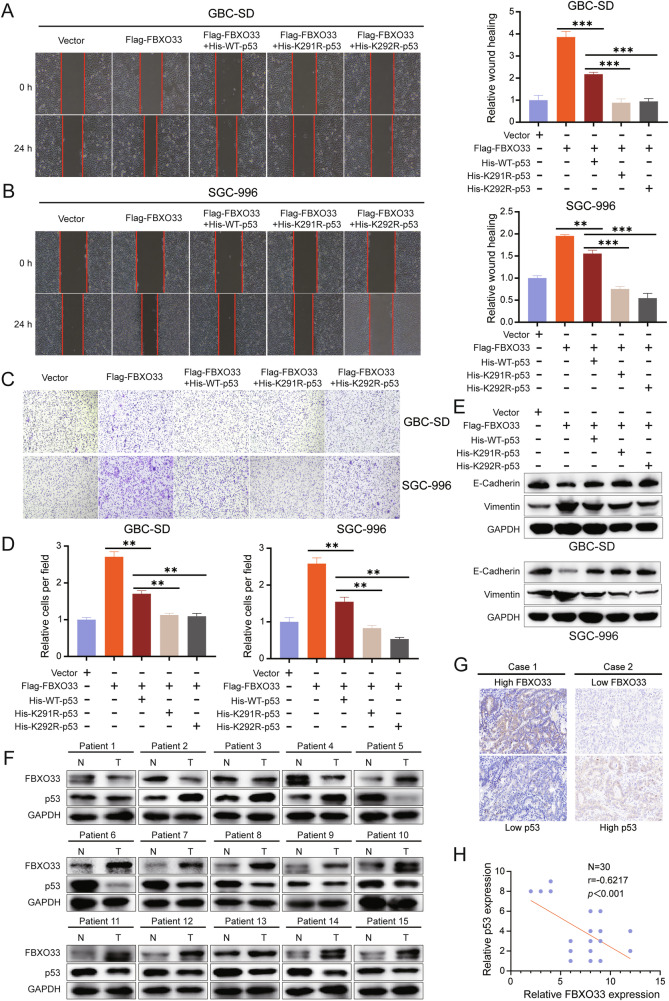
p53 regulates the function of FBXO33 in GBC and is negatively correlated with FBXO33 expression. **A**, **B** Scratch assay assessed the change in migration ability of GBC cells after transfection of His-WT-p53, His-K291R-p53, and His-K292R-p53 into Flag-FBXO33 group cells. **C**, **D** Transwell chamber assay examined the change in migration ability of GBC cells after transfection of His-WT-p53, His-K291R-p53, and His-K292R-p53 into Flag-FBXO33 group cells. **E** Western blot detected changes in E-Cadherin and Vimentin protein levels in GBC cells after transfection of His-WT-p53, His-K291R-p53, and His-K292R-p53 into Flag-FBXO33 group cells. **F** Western blot examined the expression of FBXO33 and p53 in 15 cases of GBC tissues and corresponding adjacent noncancerous tissues. **G** IHC analysis of FBXO33 and p53 expression in 30 cases of GBC tissues from the same batch. **H** Pearson correlation analysis of FBXO33 protein expression and p53 protein expression in 30 cases of GBC from the same batch. Error bars represent the mean (*n* = 3) ±SEM. **P < 0.01, ***P < 0.001.

To assess whether p53 contributes to FBXO33-mediated regulation of GBC cell migration and EMT, His-WT-p53, His-K291R-p53, and His-K292R-p53 were transfected into Flag-FBXO33 group cells. Scratch assays revealed that cell migration was significantly reduced in the Flag-FBXO33+His-WT-p53 group compared to the Flag-FBXO33 group. Further reductions in migration were observed in the Flag-FBXO33+His-K291R-p53 and Flag-FBXO33+His-K292R-p53 groups compared to the Flag-FBXO33+His-WT-p53 group (Fig. 6A, B). Similar trends were observed in Transwell assays (Fig. 6C, D). Western blot analysis showed increased E-Cadherin expression and decreased Vimentin expression in the Flag-FBXO33+His-WT-p53 group compared to the Flag-FBXO33 group. These changes were further amplified in the Flag-FBXO33+His-K291R-p53 and Flag-FBXO33+His-K292R-p53 groups compared to the Flag-FBXO33+His-WT-p53 group (Fig. 6E). These findings suggest that His-WT-p53 reduces the promotive effects of Flag-FBXO33 on GBC cell migration and EMT. Mutations at K291 and K292 (His-K291R-p53 and His-K292R-p53) further attenuated the inhibitory effects of Flag-FBXO33 on p53, resulting in significantly enhanced anti-cancer activity of p53 in these mutant groups compared to His-WT-p53. In conclusion, FBXO33 regulates GBC cell migration and EMT through p53, with K291 and K292 ubiquitination sites of p53 playing critical roles in this process.

The experimental results confirm that FBXO33 regulates GBC cell migration and EMT through p53. To further validate this conclusion, Western blot was used to analyze the protein expression levels of FBXO33 and p53 in 15 GBC patients, regardless of the mutational status of p53. In 11 cases, FBXO33 was highly expressed in cancer tissues compared to adjacent tissues, while p53 was more highly expressed in adjacent tissues than in cancer tissues. Conversely, in 3 cases, FBXO33 was more highly expressed in adjacent tissues, while p53 was more highly expressed in cancer tissues (Fig. 6F). IHC analysis of 30 consecutive GBC pathological sections revealed that high expression of FBXO33 in GBC tissues corresponded to low p53 expression, and vice versa (Fig. 6G). Correlation analysis indicated a negative relationship between the expression of FBXO33 and p53 (Fig. 6H). When examining the mutational status of p53 in 82 GBC patients (Fig. 1C), Kaplan-Meier curves showed that patients with wtp53 had higher overall survival rates compared to those with mutp53 (Supplementary Fig. S4A). These findings align with the results reported by Sunwang Xu et al. [22]. Correlation analysis revealed a negative association between FBXO33 and wtp53 expression (Supplementary Fig. S4B), but no correlation between FBXO33 and mutp53 expression (Supplementary Fig. S4C). Moreover, high FBXO33 expression was significantly associated with poor prognosis in patients with wtp53 but showed no association with prognosis in patients with mutp53 (Supplementary Fig. S4D, E). These results suggest that wtp53 regulates the function of FBXO33 in GBC and is negatively correlated with its expression.

### Transcription factor YY1 promotes FBXO33 expression in GBC

**Fig. 7:**
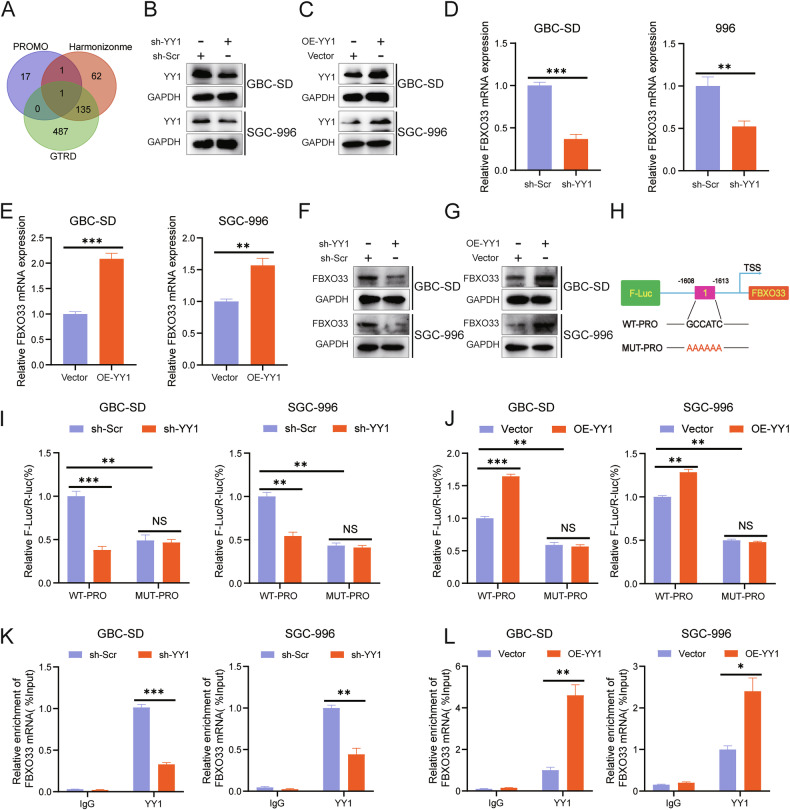
Transcription factor YY1 promotes FBXO33 expression in GBC. **A** Bioinformatics predicted YY1 as a potential transcription factor of FBXO33. **B** Western blot detected the knockdown effect of sh-YY1. **C** Western blot examined the overexpression effect of OE-YY1. RT-qPCR detected the impact of sh-YY1 (**D**) and OE-YY1 (**E**) on FBXO33 mRNA levels in GBC cells. Western blot assessed the effect of sh-YY1 (**F**) and OE-YY1 (**G**) on FBXO33 protein levels in GBC cells. **H** Schematic diagram of potential binding sequences of YY1 to the FBXO33 promoter region (WT-PRO and MUT-PRO plasmids). WT-PRO and MUT-PRO plasmids were transfected into corresponding GBC cells, and after 48 h, a dual-luciferase reporter assay was conducted to assess the effect of sh-YY1 (**I**) and OE-YY1 (**J**) on fluorescence values in WT-PRO group and MUT-PRO group. ChIP-qPCR detected the enrichment of YY1 in the FBXO33 promoter region in sh-YY1 (**K**) and OE-YY1 (**L**) groups. Error bars represent the mean (*n* = 3) ±SEM. *P < 0.05, **P < 0.01, ***P < 0.001.

The results demonstrate that FBXO33 is significantly upregulated in GBC tissues compared to adjacent tissues and regulates EMT progression, indicating its potential as a therapeutic target for GBC. To explore the regulatory mechanism of FBXO33 expression, the promoter sequence of the human FBXO33 gene (2000 bp upstream of the transcription start site) was obtained from the NCBI database. Potential transcription factors for FBXO33 were predicted using the PROMO, Harmonizome, and GTRD databases. When the PROMO database was set to 0% Maximum matrix dissimilarity rate, YY1 was identified as the only intersecting transcription factor across all three databases (Fig. 7A).

To evaluate whether YY1 regulates FBXO33 expression in GBC, GBC cells with YY1 knockdown (sh-YY1) and overexpression (OE-YY1) were constructed (Fig. 7B, C). RT-qPCR analysis revealed that sh-YY1 significantly reduced FBXO33 mRNA expression (Fig. 7D), while OE-YY1 significantly increased it (Fig. 7E). Western blot results confirmed similar effects at the protein level (Fig. 7F, G). To further investigate, a wild-type plasmid (WT-PRO) containing the potential YY1 binding sequence in the FBXO33 promoter and a mutant plasmid (MUT-PRO) with a mutated binding sequence were constructed (Fig. 7H). Dual-luciferase reporter assays demonstrated that fluorescence intensity in the WT-PRO group was higher than in the MUT-PRO group (Fig. 7I, J). Furthermore, sh-YY1 reduced fluorescence intensity in the WT-PRO group but had no significant effect on the MUT-PRO group (Fig. 7I). Conversely, OE-YY1 increased fluorescence intensity in the WT-PRO group but had no significant effect on the MUT-PRO group (Fig. 7J). ChIP-qPCR analysis showed that YY1 was enriched in the FBXO33 promoter region. Knockdown of YY1 significantly reduced enrichment, while overexpression of YY1 significantly increased it (Fig. 7K, L). These results confirm that in GBC, YY1 promotes the transcriptional activation of FBXO33.

### Transcriptional activation of FBXO33 by YY1 in GBC depends on YY1 lactylation modification

Protein lactylation modification (Kla) is a recently identified post-translational modification that plays a vital role in regulating gene expression [25]. Notably, YY1 is one of the proteins susceptible to lactylation modification [26], with the lactylation site K183 playing a critical role in its transcriptional function [27]. Based on this, it was hypothesized that the transcriptional activation of FBXO33 by YY1 is also regulated by lactylation modification.

**Fig. 8:**
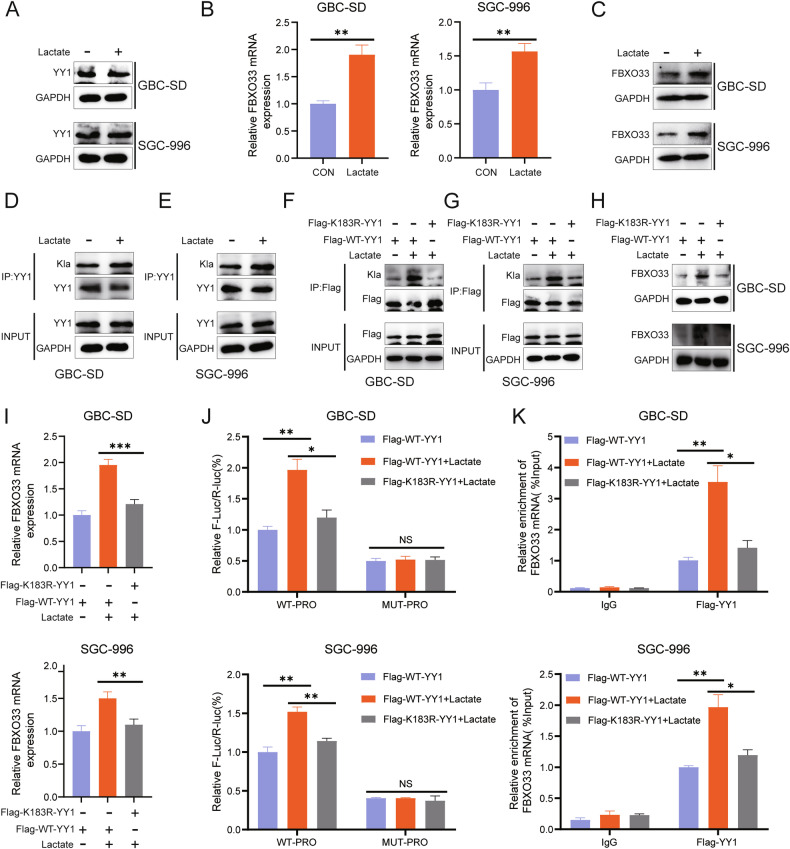
YY1-dependent transcriptional activation of FBXO33 in GBC depends on YY1 lactylation modification. **A** Western blot detected the effect of lactate (25 mM) on YY1 protein. RT-qPCR and Western blot detected the effect of lactate on FBXO33 mRNA (**B**) and FBXO33 protein (**C**). **D**, **E** Co-IP detected the effect of lactate on YY1 protein lactylation modification. **F**, **G** Co-IP detected the effect of lactate on YY1 protein lactylation modification after mutation of YY1 protein K183 lactylation modification site. Western blot and RT-qPCR detected the effect of lactate on FBXO33 protein (**H**) and FBXO33 mRNA (**I**) after mutation of YY1 protein K183 lactylation modification site. **J** WT-PRO and MUT-PRO plasmids were transfected into Flag-WT-YY1 and Flag-K183R-YY1, and after 48 h, a dual-luciferase reporter assay was conducted to assess the effect of lactate on fluorescence values in each group. **K** ChIP-qPCR detected the effect of lactate on the enrichment of YY1 in the FBXO33 promoter region in Flag-WT-YY1 group and Flag-K183R-YY1 group. Error bars represent the mean (*n* = 3) ±SEM. *P < 0.05, **P < 0.01, ***P < 0.001.

To test this hypothesis, experiments were conducted. RT-qPCR and Western blot analysis showed that exogenous lactate did not alter the protein expression of YY1 (Fig. 8A) but significantly increased the mRNA and protein expression of FBXO33 (Fig. 8B, C). Co-IP experiments demonstrated that lactate enhanced the lactylation level of YY1 (Fig. 8D, E). Since YY1 has only one lactylation site (K183) [27], lysine K183 was mutated to arginine, creating a wild-type YY1 plasmid (Flag-WT-YY1) and a K183 mutant plasmid (Flag-K183R-YY1). Co-IP results indicated that mutation of the K183 lactylation site reduced the lactylation level of YY1 (Fig. 8F, G).

Further, Western blot analysis revealed that in the Flag-K183R-YY1 group, exogenous lactate failed to increase FBXO33 mRNA and protein expression, unlike in the Flag-WT-YY1 group (Fig. 8H, I). Dual-luciferase reporter assays confirmed that lactate significantly enhanced fluorescence intensity in the WT-PRO group but had no significant effect on the MUT-PRO group (Fig. 8J). After K183 mutation, lactate could no longer increase fluorescence intensity in the WT-PRO group (Fig. 8J). ChIP-qPCR analysis showed that lactate significantly elevated the enrichment level of YY1 at the FBXO33 promoter region (Fig. 8K), but this effect was lost after K183 mutation (Fig. 8K). In conclusion, the K183 lactylation modification of YY1 enhances its ability to bind to the FBXO33 promoter region, thereby regulating the transcriptional activation of FBXO33.

## Discussion

In recent years, studies have increasingly highlighted the critical role of protein ubiquitination in tumor development and progression [28, 29]. Targeting ubiquitin signaling pathways presents significant potential for cancer therapy [20, 30]. A detailed understanding of ubiquitination targets and their specific mechanisms in GBC could serve as a foundation for developing novel therapeutic strategies. Among E3 ubiquitin ligases, the F-box family has emerged as a key regulator of EMT progression and holds promise for cancer treatment [31, 32]. For instance, FBXO43, which is highly expressed in various malignancies, promotes oncogenic activity by stabilizing SKP2, a cell cycle regulator [33]. Similarly, FBXO45 facilitates pancreatic cancer progression by regulating the stability of the tumor suppressor USP49 [34]. FBXO33, an E3 ubiquitin ligase associated with Cullin 1 [35], is a member of the F-box protein family, characterized by uncharacterized domains [31, 36]. Experimental results in this study confirmed that FBXO33 is upregulated in GBC and positively correlates with distant metastasis and TNM staging. Knockdown of FBXO33 inhibits EMT progression in GBC, while its overexpression promotes EMT. These findings demonstrate that FBXO33 functions as an oncogene in GBC.

E3 ubiquitin ligases link target proteins with specific E2 ubiquitin-conjugating enzymes [8, 37], modulating the biological functions of their substrates [38]. However, limited studies have explored the target proteins of FBXO33, with only EIF3F [39],YBX1 [40], and MYC [15] confirmed as its ubiquitination targets. p53, one of the first identified tumor suppressors [41], has been extensively studied in cancer research [42, 43]. When mutated, p53 loses its tumor-suppressive functions, and mutp53 gains oncogenic properties, promoting cancer cell survival, proliferation, and metastasis [44]. Mutations in p53 are common in GBC [45] and are associated with poorer patient prognoses [22]. The ubiquitination of p53 has been extensively studied in recent years. MDM2, a well-known ubiquitin ligase, promotes p53 ubiquitination and represents a promising target for cancer therapy through the MDM2/p53 axis [46, 47]. Additionally, p53 has been identified as a substrate for FBXL8 [48], VPRBP [49], TRIM31 [50], and SCML2 [51]-mediated ubiquitination. Interestingly, both wtp53 and mutp53 can undergo ubiquitination [52], with some E3 ubiquitin ligases targeting both forms [53, 54], while others exhibit specificity for either wtp53 or mutp53 [55–57]. In this study, p53 was predicted to be a ubiquitination target of FBXO33 through the Ubibrowser platform and subsequently confirmed by Co-IP experiments. Further analysis revealed that FBXO33’s effect on wtp53 is significantly stronger than on mutp53. Mechanistic studies identified the DNA binding domain (DBD) of p53 as the critical region for FBXO33-mediated ubiquitination, with K291 and K292 in the DBD playing essential roles in regulating GBC EMT progression. Analysis of tissue samples from GBC patients further confirmed the correlation between FBXO33 and wtp53, but not mutp53. It was also observed that high expression of FBXO33 is negatively correlated with prognosis in patients with wtp53, whereas no such correlation was observed in patients with mutp53.

The transcription factor YY1 regulates the expression of various genes in malignant tumors by mediating enhancer-promoter interactions [58, 59]. Using predictions from multiple databases, potential binding sequences of YY1 were identified in the promoter region of FBXO33. Experimental verification confirmed that YY1 acts as a transcription factor for the FBXO33 gene, partially explaining the high expression of FBXO33 in GBC. In the context of protein post-translational modification, protein lactylation induced by tumor metabolite lactate plays a critical role in regulating protein function [60]. The K183 site on YY1 is a key lactylation site [26, 27]. Dual-luciferase reporter assays and ChIP-qPCR analysis demonstrated that lactate enhances the enrichment of YY1 in the promoter region of FBXO33. Further experiments confirmed that the binding ability of YY1 to the FBXO33 promoter region is strengthened following lactylation modification at K183, revealing a novel mechanism for the transcriptional activation of FBXO33 in GBC.

In conclusion, this study highlights the positive correlation between FBXO33 expression and the prognosis of GBC. As an oncogene, FBXO33 promotes EMT progression by enhancing p53 ubiquitination at K291 and K292, leading to decreased p53 expression. Furthermore, YY1 activates FBXO33 transcription in GBC through K183 lactylation modification, sustaining high FBXO33 expression in GBC. These findings elucidate the mechanism of action of FBXO33 in GBC and provide potential strategies for targeted ubiquitin signaling therapy in GBC.

## Supplementary information

Supplementary Information

original western blot

## Data Availability

The datasets used and analyzed during the current study are available from the corresponding author on reasonable request.
