# Supplementary material for: Lactylation-driven transcriptional activation of FBXO33 promotes gallbladder cancer metastasis by regulating p53 polyubiquitination
Source: Cell Death Dis. 2025 Feb 28;16(1):144. doi: 10.1038/s41419-025-07372-y (PMC11871038; doi:10.1038/s41419-025-07372-y)
Supplement: Supplementary file 1 — Supplementary Information [file 41419_2025_7372_MOESM1_ESM.pdf]

Supplementary Figure

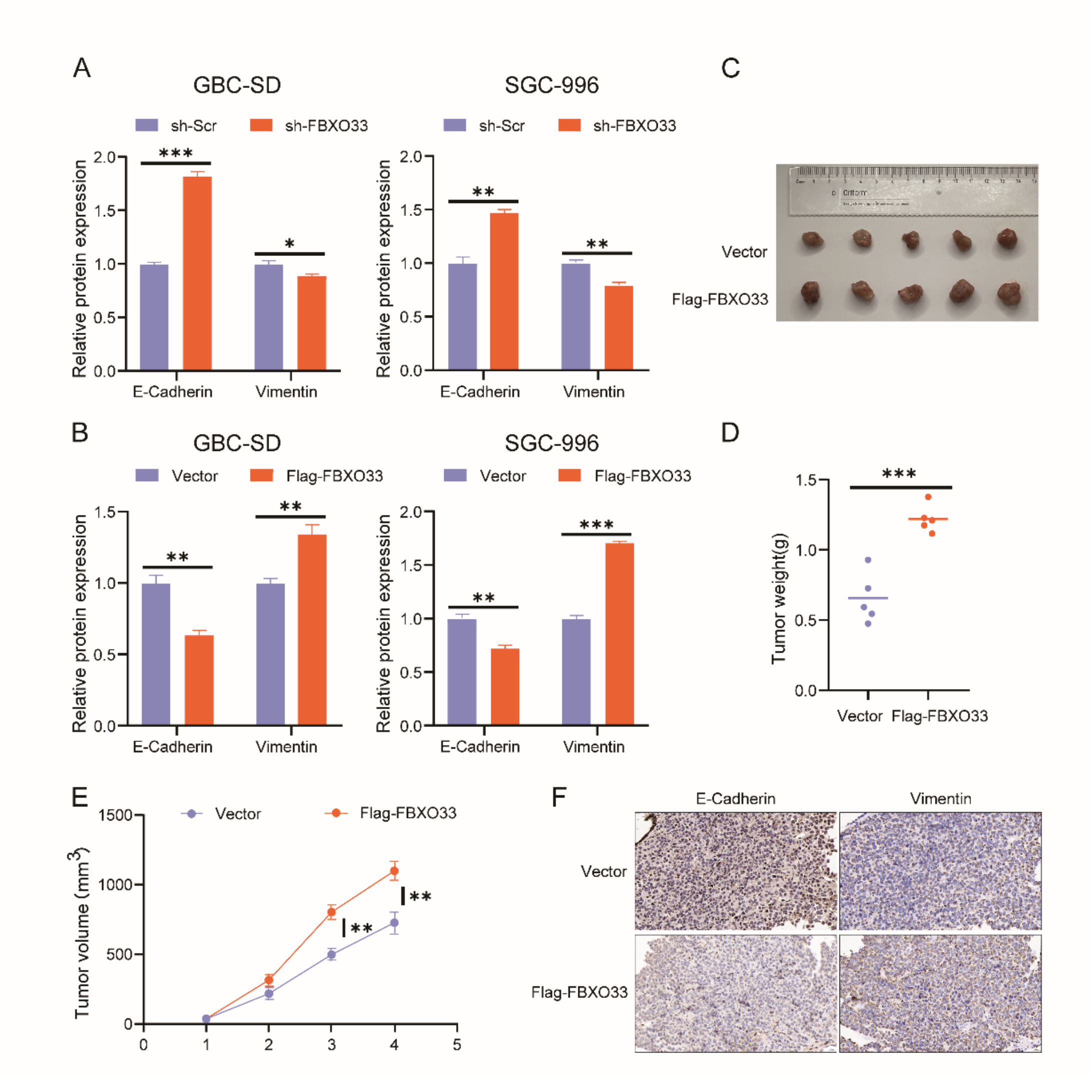

Figure S1. FBXO33 regulates EMT progression of GBC in vitro and in vivo. (A) Quantitative plots depicting changes in E-Cadherin and Vimentin protein levels following FBXO33 knockdown in GBC cells. (B) Quantitative plots illustrating alterations in E-Cadherin and Vimentin protein levels upon FBXO33 overexpression in GBC cells. (C) Impact of Flag-FBXO33 on subcutaneous tumor size in GBC. (D-E) Impact of Flag-FBXO33 on subcutaneous tumor (D) weight and (E) volume in GBC. (L) IHC analysis of the effect of Flag-FBXO33 on the protein expression of E-Cadherin and Vimentin in GBC subcutaneous tumors. Error bars represent the mean (n=3) ± SEM.

*$p<0.05$，**$p<0.01$，***$p<0.001$.

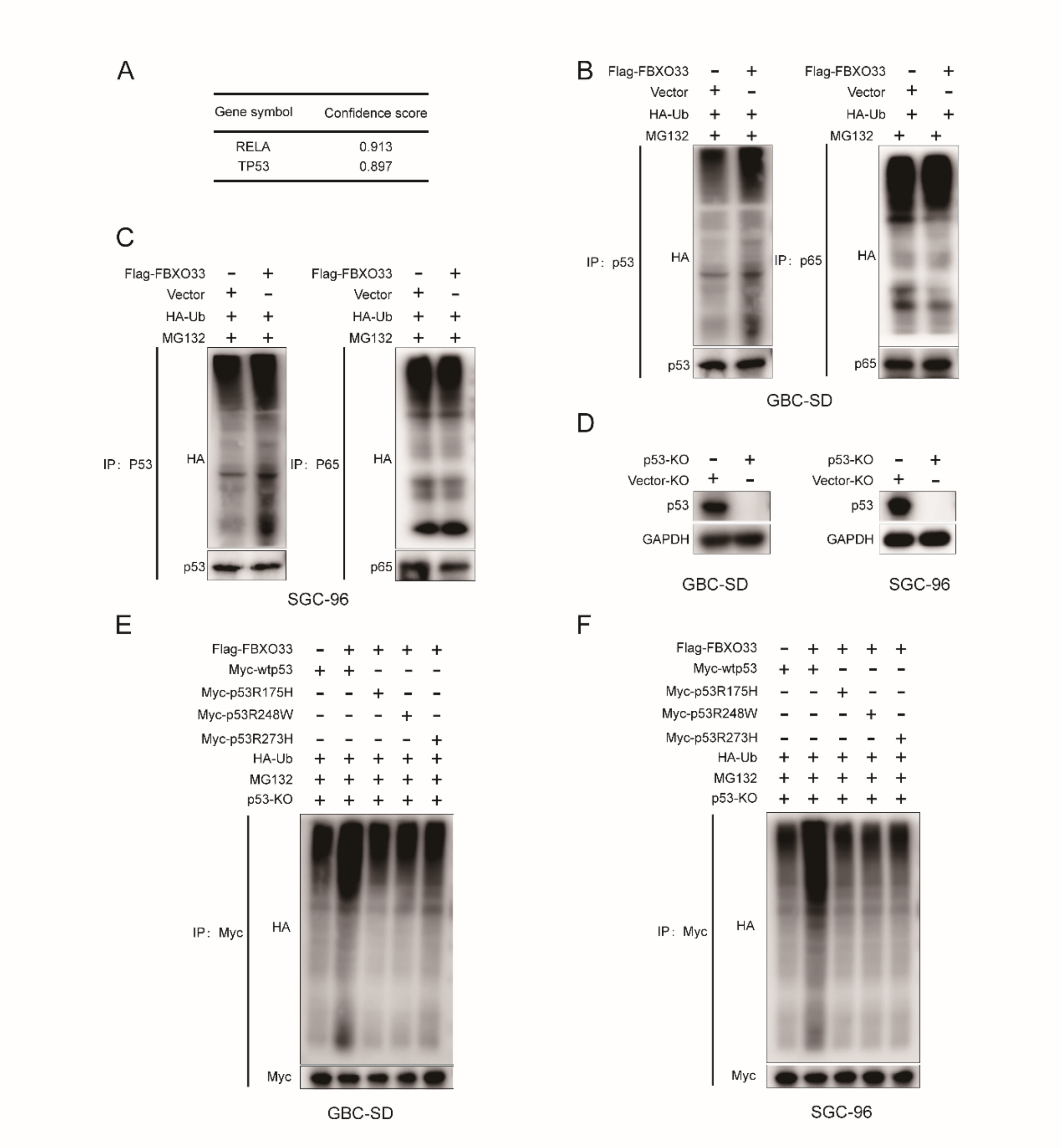

Figure S2. Interaction between FBXO33 and wtp53. (A) UbiBrowser identified RELA and TP53 as the substrates of FBXO33 with the highest confidence scores, providing detailed specific scores for each molecule. (B-C) Co-IP detected the effect of Flag-FBXO33 on the ubiquitin binding of p53 and p65. (D) Western blot analysis was conducted to assess the knockdown efficacy of p53 in GBC cells. (E-F) Co-IP assays were performed to investigate the impact of Flag-FBXO33 on the ubiquitination of wtp53 and mutp53 in GBC cells with p53 knockout.

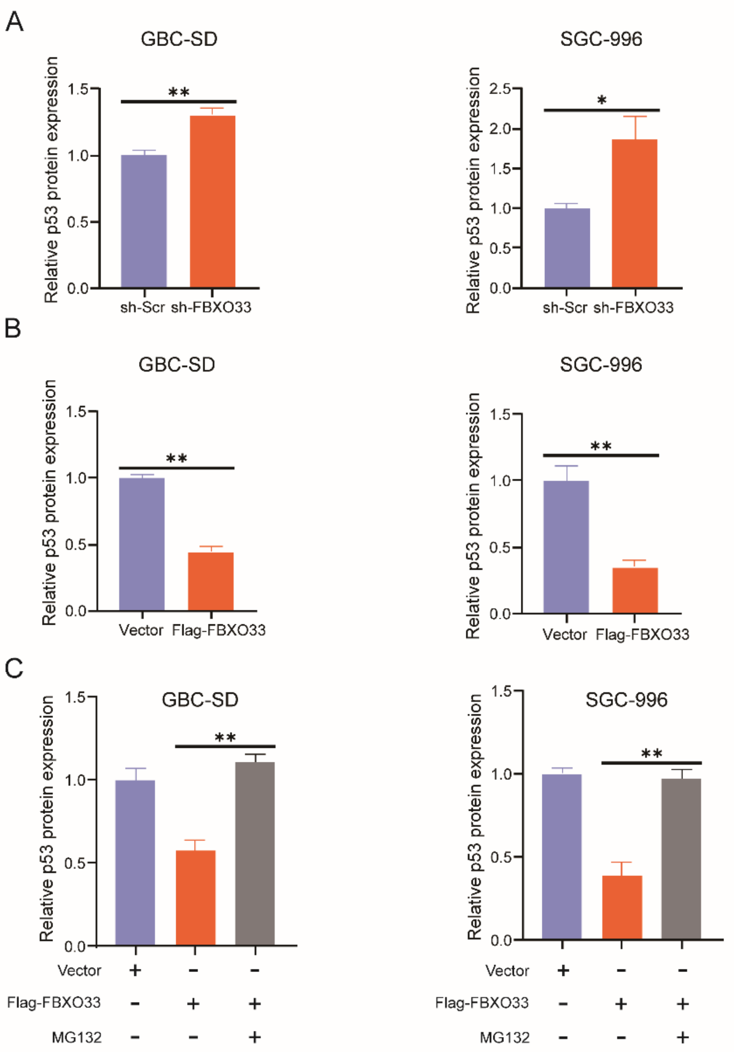

Figure S3. FBXO33 regulates the stability of p53 protein. (A) Quantitative plots depicting changes in p53 protein levels following FBXO33 knockdown in GBC cells. (B) Quantitative plots illustrating alterations in p53 protein levels upon FBXO33 overexpression in GBC cells. (C) Quantitative analysis was performed to evaluate the effect of MG132 on the mediated degradation of p53 protein by FBXO33. Error bars represent the mean (n=3) ± SEM. *$p<0.05$, **$p<0.01$.

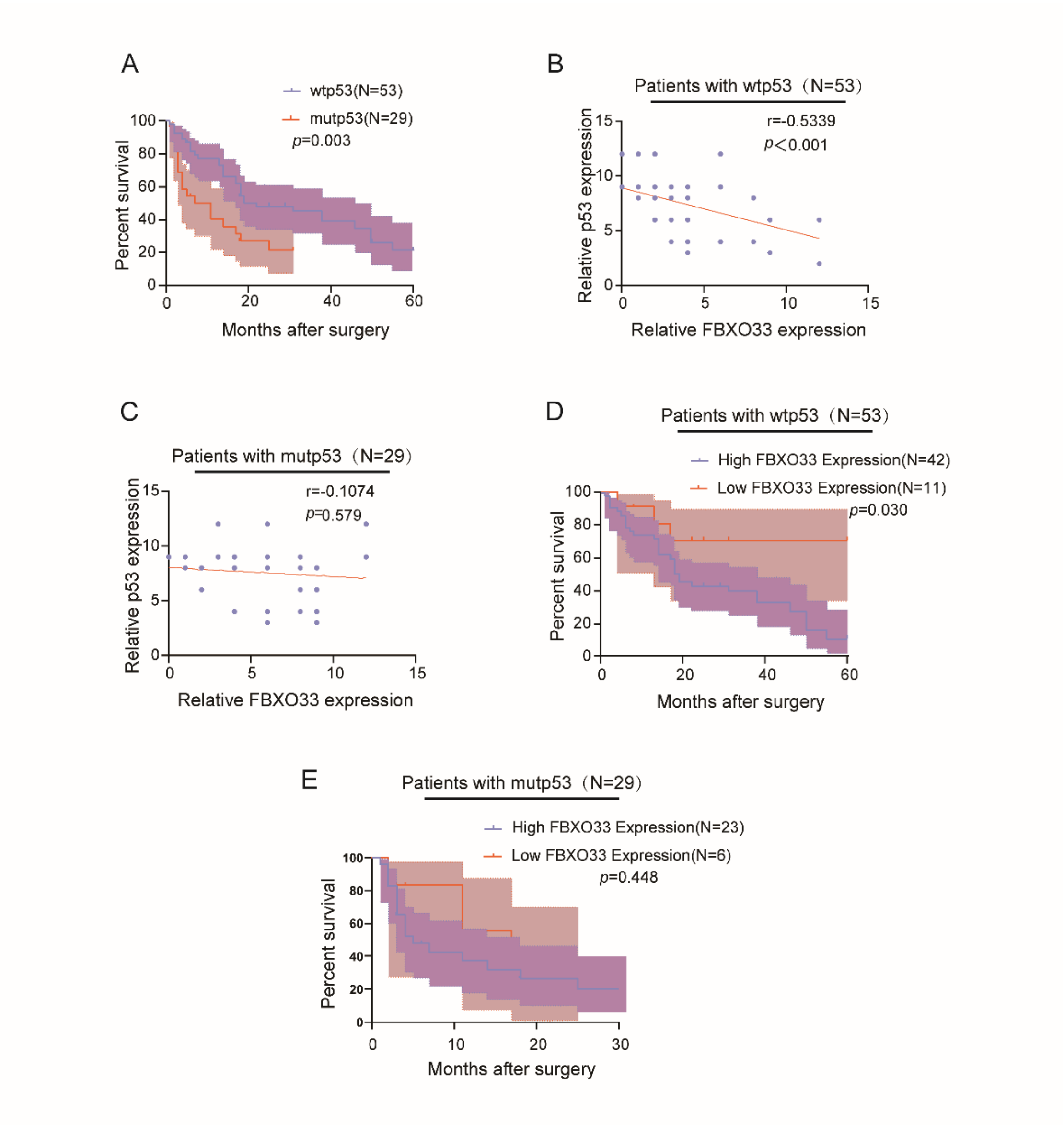

Figure S4. High expression of FBXO33 was found to be negatively associated with poor prognosis in GBC patients with wild-type p53. (A) Relationship between p53 mutation status and overall survival rate of GBC patients. (B-C) Pearson correlation analysis was conducted to assess the relationship between FBXO33 protein expression and p53 protein expression in GBC patients with wtp53(B) and mutp53(C). (D-E) Relationship between FBXO33 protein expression and overall survival rate of GBC patients with wtp53(D) and mutp53(E).
