# Supplementary figures and images for: Lactylation-driven transcriptional activation of FBXO33 promotes gallbladder cancer metastasis by regulating p53 polyubiquitination
Source: Cell Death Dis. 2025 Feb 28;16(1):144. doi: 10.1038/s41419-025-07372-y (PMC11871038; doi:10.1038/s41419-025-07372-y)

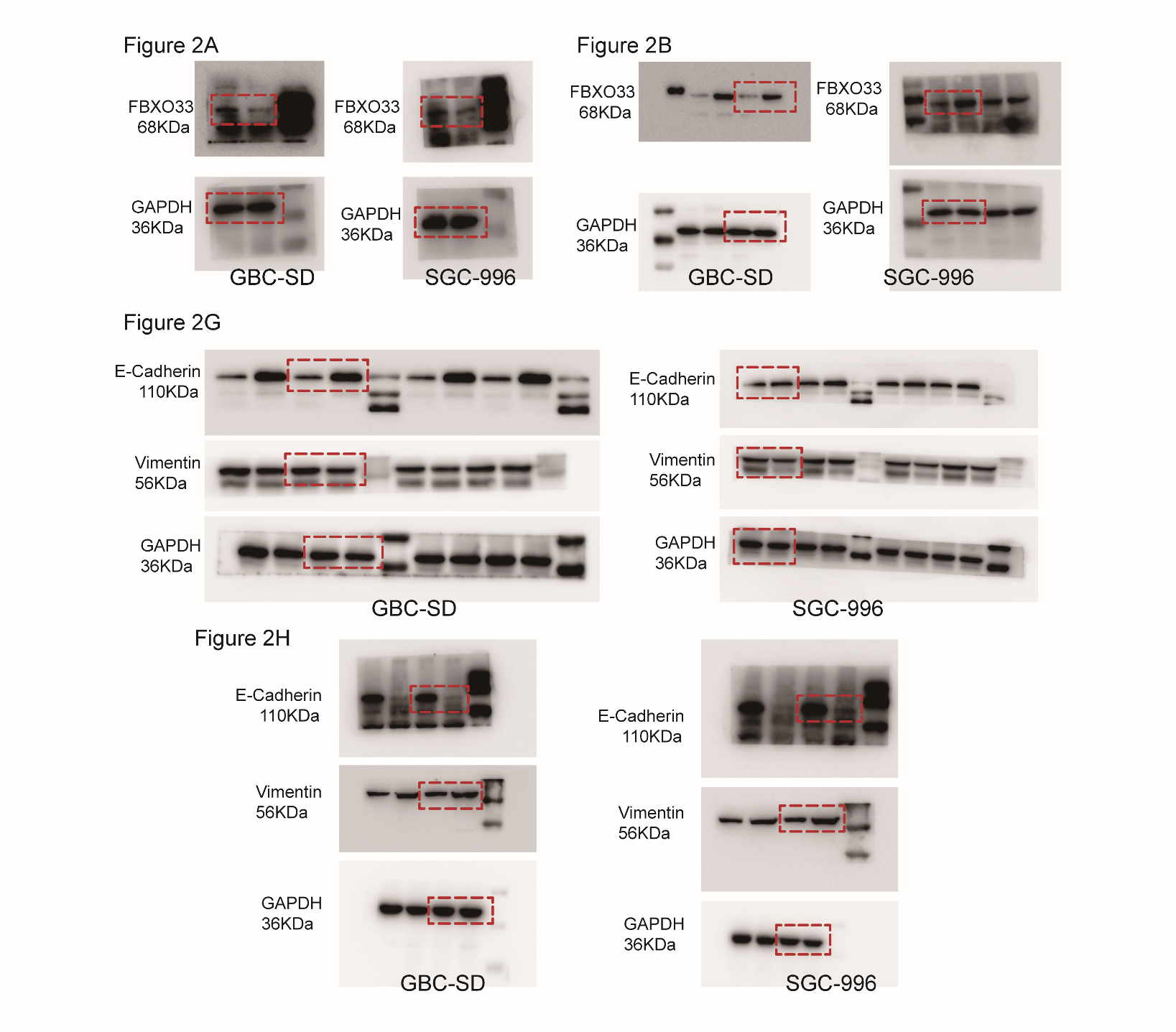


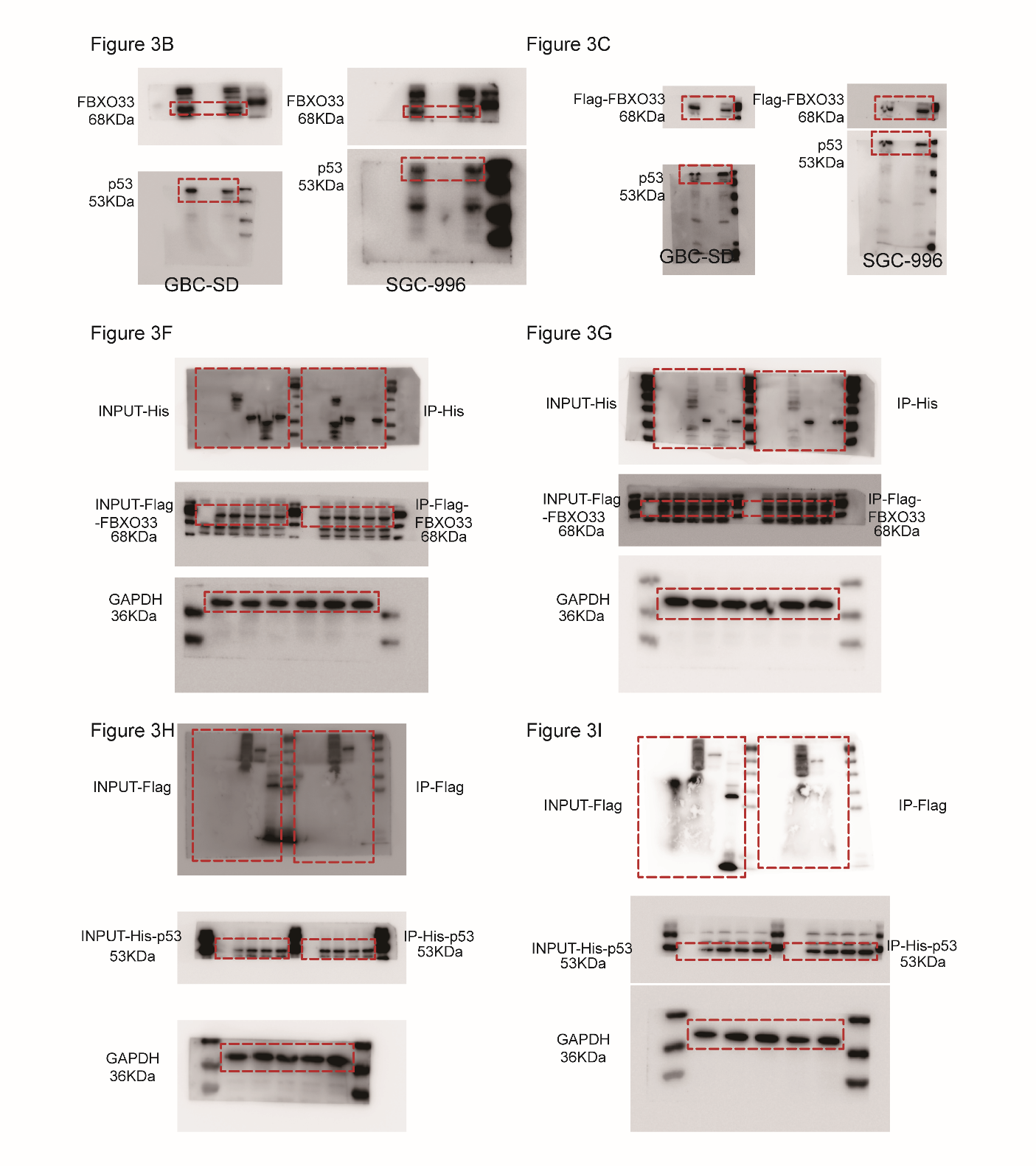


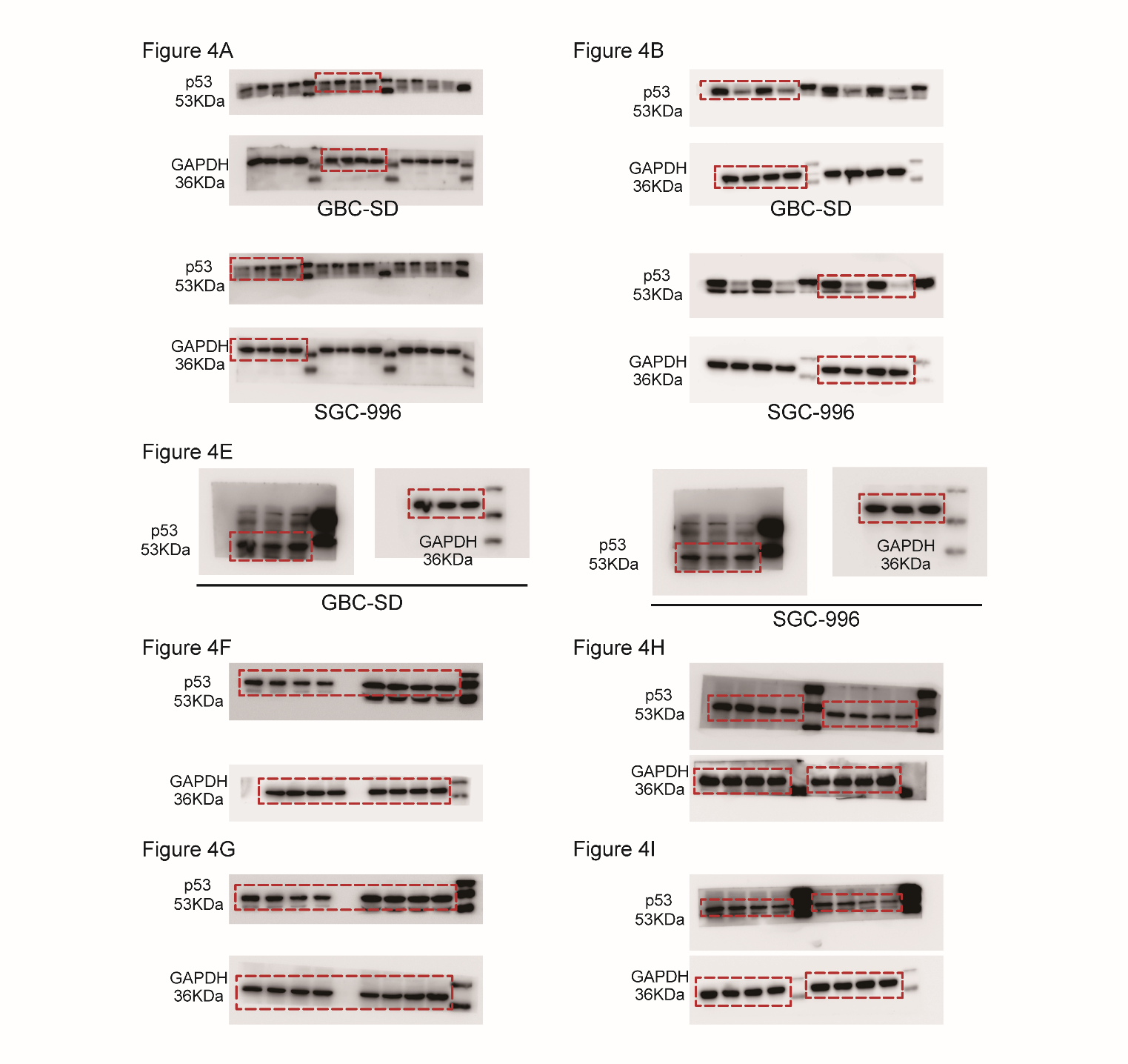


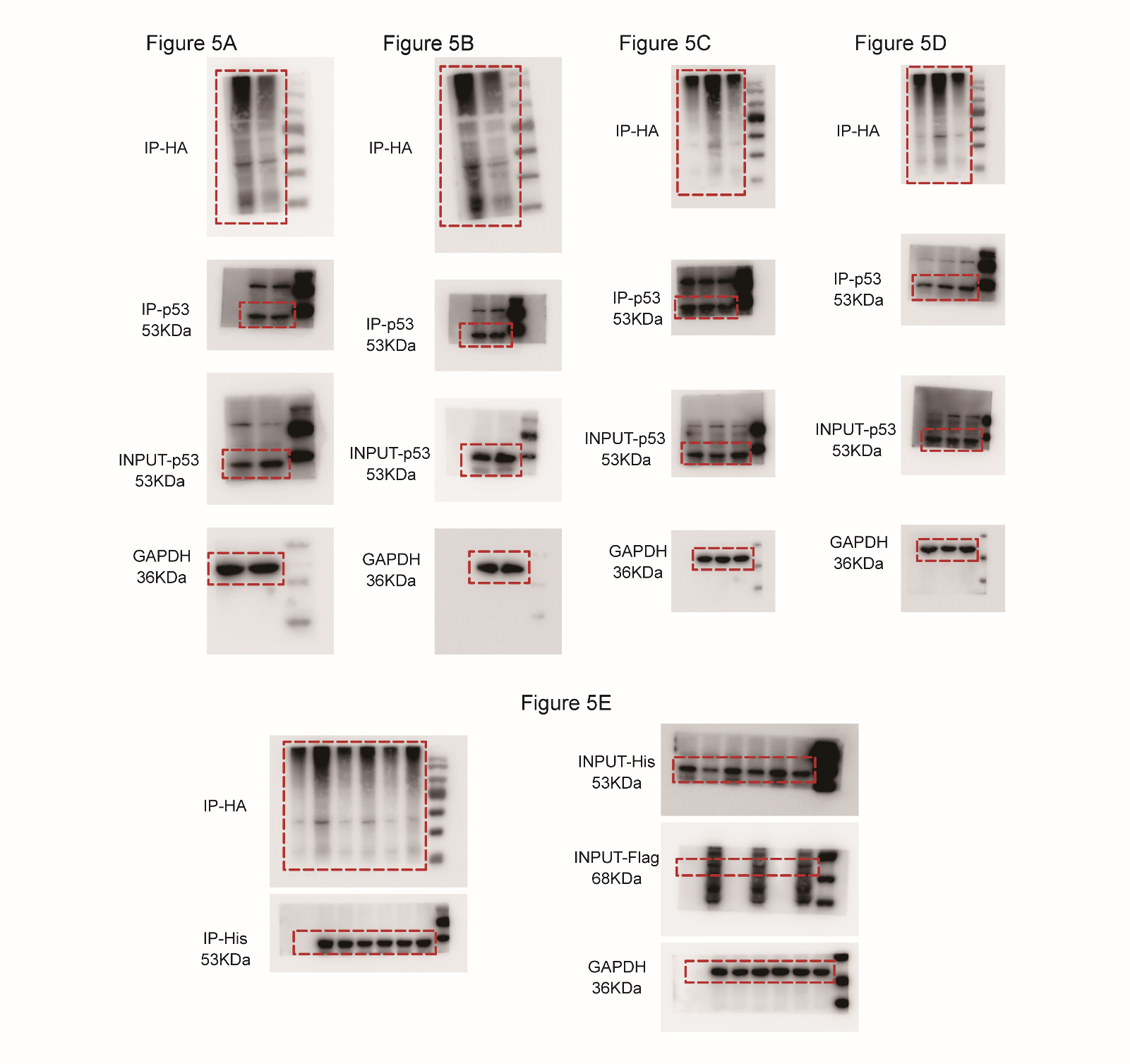


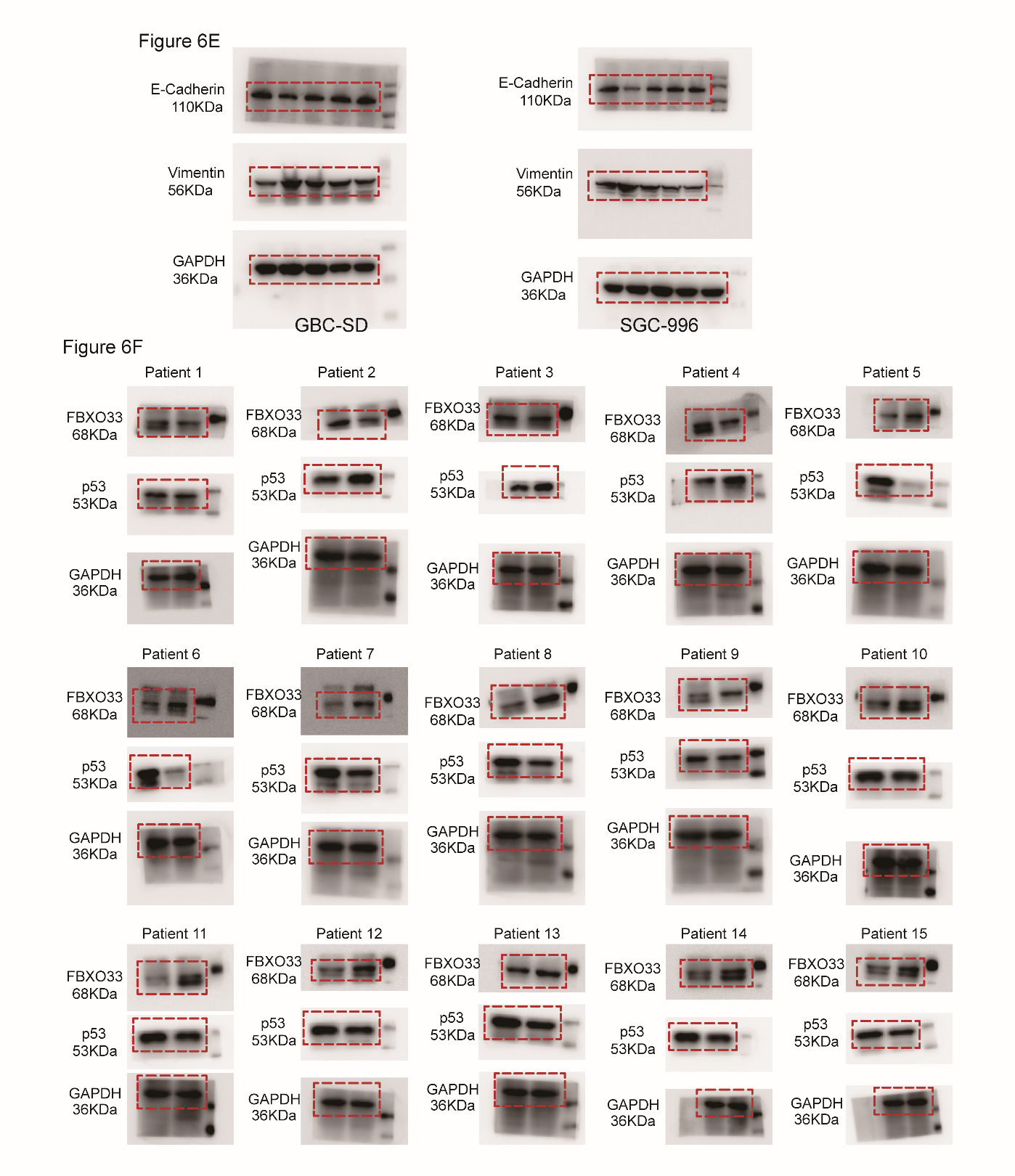


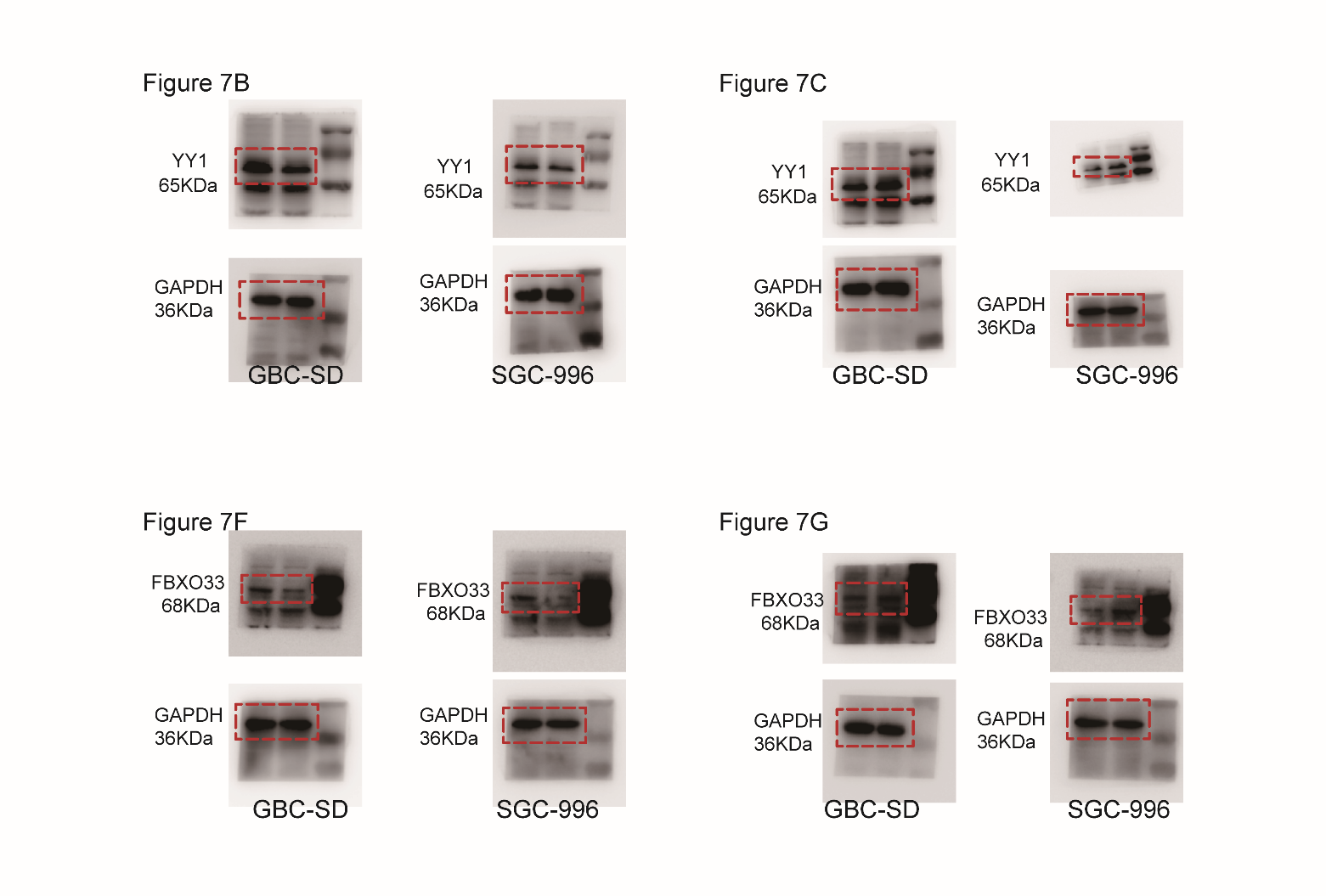


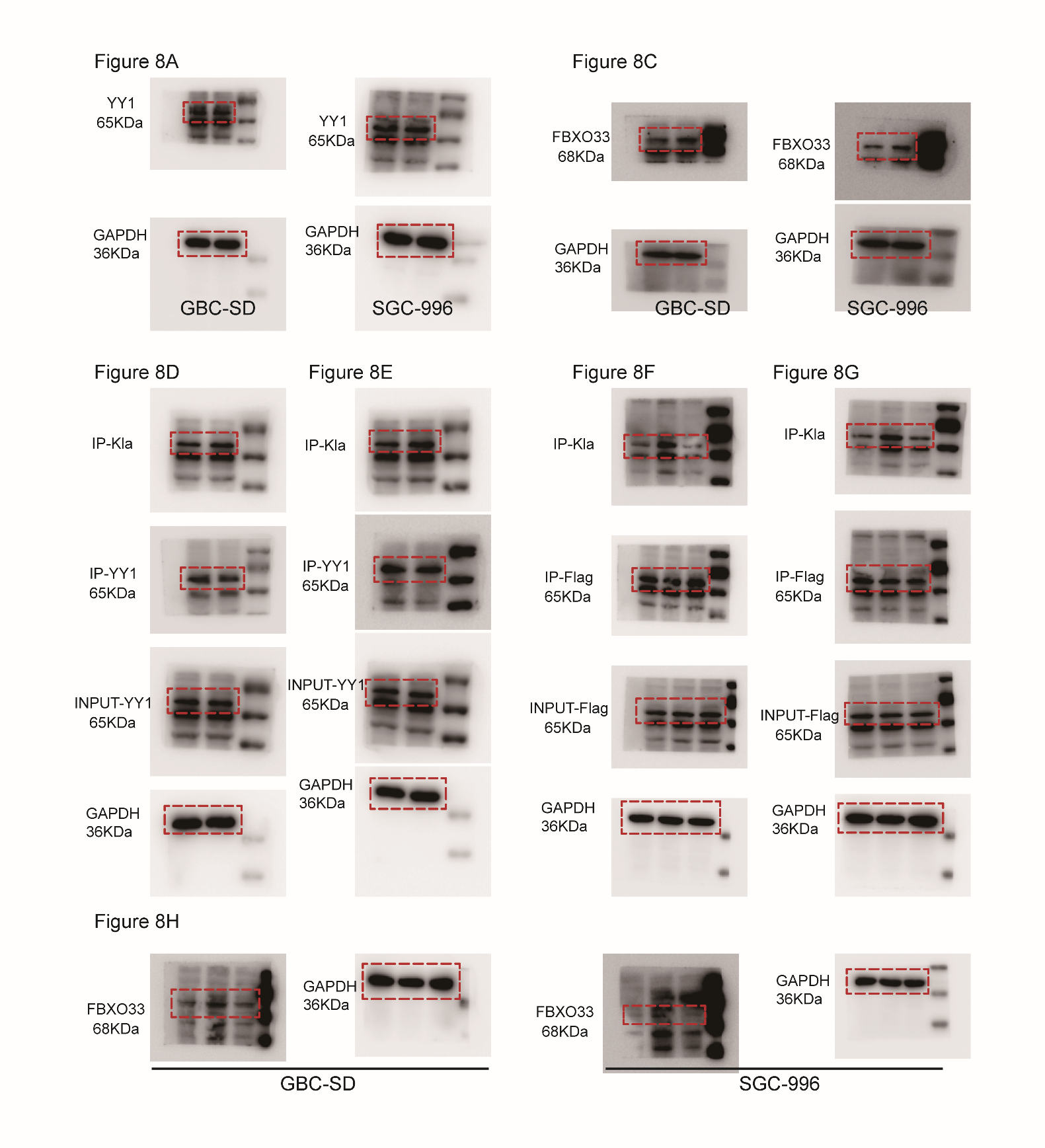


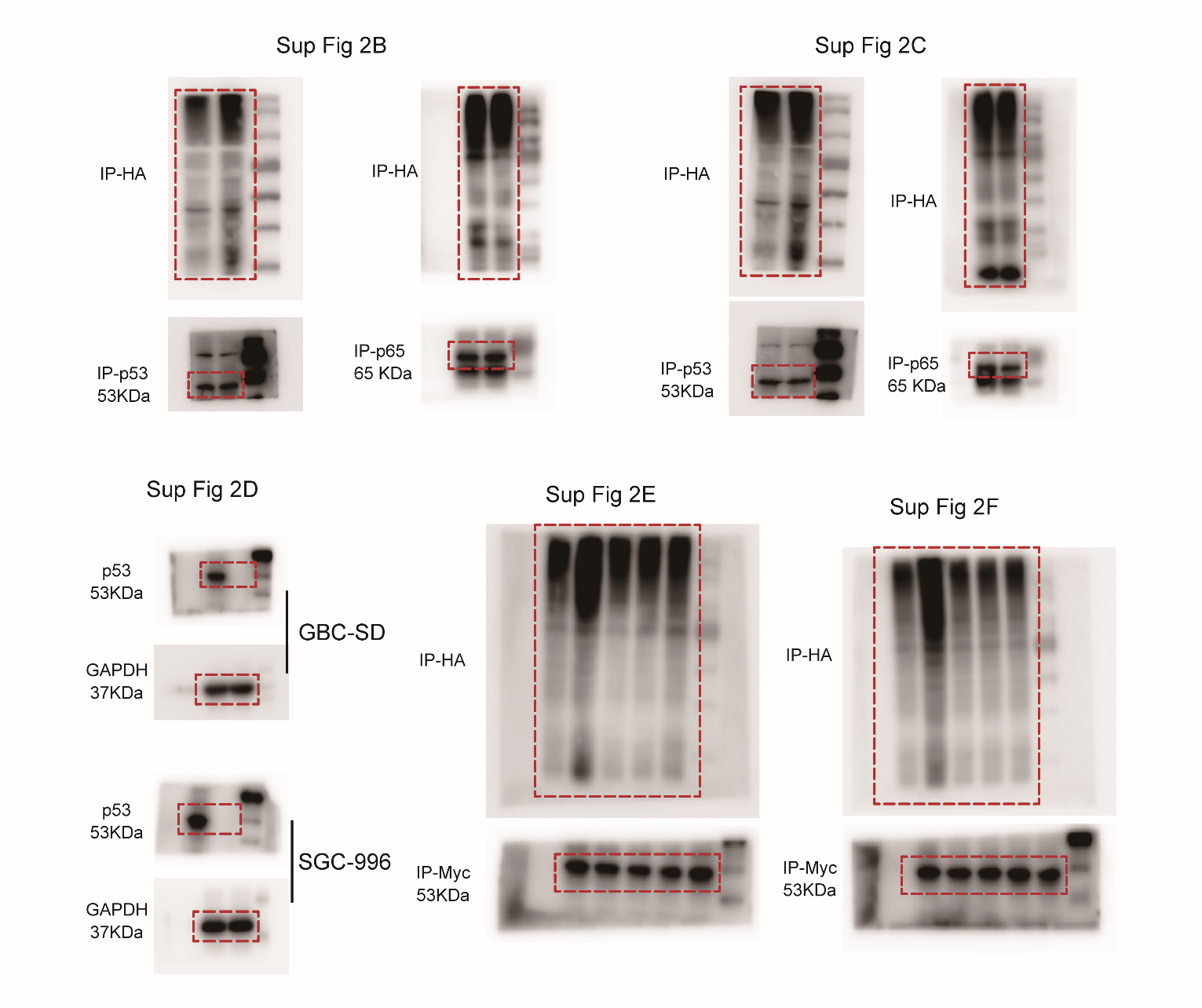

Supplement: Supplementary file 2 — original western blot [file 41419_2025_7372_MOESM2_ESM.docx]
